# Impacts of Human Recreation on Brown Bears (*Ursus arctos*): A Review and New Management Tool

**DOI:** 10.1371/journal.pone.0141983

**Published:** 2016-01-05

**Authors:** Jennifer K. Fortin, Karyn D. Rode, Grant V. Hilderbrand, James Wilder, Sean Farley, Carole Jorgensen, Bruce G. Marcot

**Affiliations:** 1 US Geological Survey, Alaska Science Center Anchorage, Alaska, United States of America; 2 National Park Service – Alaska Region, Anchorage, Alaska, United States of America; 3 US Fish and Wildlife Service, Anchorage, Alaska, United States of America; 4 Alaska Department of Fish & Game, Anchorage, Alaska, United States of America; 5 Chugach National Forest, USDA Forest Service, Anchorage, Alaska, United States of America; 6 Pacific Northwest Research Station, USDA Forest Service, Portland, Oregon, United States of America; 7 College of Forestry and Conservation, University of Montana, Missoula, MT, United States of America; Sonoma State University, UNITED STATES

## Abstract

Increased popularity of recreational activities in natural areas has led to the need to better understand their impacts on wildlife. The majority of research conducted to date has focused on behavioral effects from individual recreations, thus there is a limited understanding of the potential for population-level or cumulative effects. Brown bears (*Ursus arctos*) are the focus of a growing wildlife viewing industry and are found in habitats frequented by recreationists. Managers face difficult decisions in balancing recreational opportunities with habitat protection for wildlife. Here, we integrate results from empirical studies with expert knowledge to better understand the potential population-level effects of recreational activities on brown bears. We conducted a literature review and Delphi survey of brown bear experts to better understand the frequencies and types of recreations occurring in bear habitats and their potential effects, and to identify management solutions and research needs. We then developed a Bayesian network model that allows managers to estimate the potential effects of recreational management decisions in bear habitats. A higher proportion of individual brown bears in coastal habitats were exposed to recreation, including photography and bear-viewing than bears in interior habitats where camping and hiking were more common. Our results suggest that the primary mechanism by which recreation may impact brown bears is through temporal and spatial displacement with associated increases in energetic costs and declines in nutritional intake. Killings in defense of life and property were found to be minimally associated with recreation in Alaska, but are important considerations in population management. Regulating recreation to occur predictably in space and time and limiting recreation in habitats with concentrated food resources reduces impacts on food intake and may thereby, reduce impacts on reproduction and survival. Our results suggest that decisions managers make about regulating recreational activities in time and space have important consequences for bear populations. The Bayesian network model developed here provides a new tool for managers to balance demands of multiple recreational activities while supporting healthy bear populations.

## Introduction

Participation in outdoor recreational activities in the United States has increased each decade since the 1950s and is predicted to continue to increase with population growth and the growing desire to experience nature [1, 2, 3]. From 2000 to 2009, the number of participants in nature-based recreation increased by 7.1% and the number of user days increased by 40% [3]. Alaska has seen particularly high increases in recreational activity. From 2001 to 2011, the annual number of anglers, hunters, and wildlife watchers (primarily bear-viewers) increased by 28%, 34%, and 71%, respectively [4]. In 2012, 89% of Canadian adults participated in nature-related activities and 57% in nature-based travel [5]. Recreational activities in wildlife habitats have important economic benefits. In the US $32.2 billion was spent by hunters and anglers in 2011 and in Canada $40.4 billion was spent on nature related activities in 2012. In the southeast United States, wildlife associated recreation was estimated to provide 783,000 jobs and $22 to $48 billion in expenditures [6].

Given the significance and economic importance of nature based recreation and its growth, it is not surprising that wildlife managers are often faced with difficult decisions regarding how to manage and support recreational opportunities while maintaining stable wildlife populations. Recreational activities can affect wildlife populations by disturbing individual animals, degrading habitat, attracting animals into conflict situations with humans as a result of improper storage of food and garbage which can lead to management removals or defense of life and property kills, and through direct mortality as a result of hunting [7, 8, 9]. About half of surveyed recreationists believed they had a negative impact on wildlife [8]. Disturbance of wildlife may also result in decreased visitor satisfaction if displacement occurs, since the species of animal viewed (particularly mega-fauna) and the number of animals viewed are directly related to the quality of a viewing experience [10, 11, 12]. The literature on disturbance of wildlife by recreational activities is relatively limited with broad information gaps, despite the potential impacts of recreation on wildlife and the need to identify mitigating management [9, 13].

Humans and the perceived risk of predation elicit similar responses from many wildlife species [14, 15, 16]. Individual animals might 1) increase vigilance at the expense of time spent in other fitness-enhancing activities (caribou [17], birds [18]) [9]; 2) expend energy to flee, limiting time in areas that may otherwise offer important resources (birds [18, 19], moose (*Alces alces*) [20]); or 3) avoid the risk altogether by foraging in potentially sub-optimal habitats [14, 15]. If human activity is limited to daytime, animals may become more nocturnal (wolves [21]). Alternatively, animals may also change their activity budgets, and associated energetic costs, rather than be displaced if there are no alternative habitats [22]. Decreased body condition and ultimately decreased reproductive success can occur if disturbance leads to decreased food intake or increased energy expenditure, theoretically having a population level impact [15, 19, 23].

Recreational activities are particularly common in bear habitats. One of the fastest growing and most commonly managed recreational activities is bear-viewing. The majority of this activity occurs along coastal Alaska where bears congregate to feed on the multiple salmon runs that occur from mid- to late summer, which also attracts anglers. However, even outside of Alaska, tourists have ranked brown bears (*Ursus arctos*) as the animal they most wish to see and viewing them resulted in higher visitor satisfaction [10]. Many recreationists participate in wildlife viewing as a secondary activity, for example, in the U.S. in 2011, 23% of hunters and anglers also participated in wildlife watching [4].

Recreational activities can lead to direct human-caused mortality of brown bears [24, 25, 26] and not just indirect behavioral effects. Bears may be removed from a habitat by lethal or non-lethal means to protect human life and property where recreationists and bears interact [27, 28, 29]. Recreational activities in bear habitats have the potential to directly and indirectly affect habitat use and survival.

In this study, we sought to better understand the potential population-level effects of recreational activities and provide a tool to aid managers in making decisions about recreational activities in brown bear habitats. We first conducted a literature review to summarize results of empirical studies on the potential effects of recreational activities on brown bears. We then used the results of the literature review to survey brown bears experts on the frequency of recreations occurring in bear habitats, their perceptions of potential effects, possible management solutions, and research needs [30, 31]. In particular, we sought expert information that has been difficult to obtain from empirical studies. For example, studies of the effects of human recreation are rarely able to deal with complex issues of cumulative effects of multiple recreational activities or the ultimate consequences of behavioral changes on individual health and population dynamics. Finally, we combined information from the literature and expert knowledge into a Bayesian network model (BNM) to aid managers when evaluating the potential impacts of human recreational activities on brown bears. Although our BNM relied heavily on expert knowledge, it provides an explicit process for aiding management decisions in situations where those decisions are currently being made much less explicitly. The BNM also allowed us to account for uncertainty in expert knowledge in the building of the model, which is also important information for managers. We limited the application of the BNM to Alaskan brown bears due to differences in the scope of recreational activities that occur within and outside of Alaska. This model will allow managers to compare the potential consequences of various management scenarios of multiple recreations in Alaskan brown bear habitat on cub survival, reproduction, and adult survival. With modification, this model could also be applied to brown bear populations in other parts of their range.

## Methods

### Literature Review

We reviewed and summarized results from peer-reviewed articles, reports, and theses containing original data on brown bears pertaining to human recreational activities. A literature search was conducted between March and December 2013 in the databases Google Scholar, Web of Science, BioOne, ScienceDirect, and Wildlife & Ecology Worldwide. We used the following key words individually and in combination with grizzly bears, bears, or brown bears: recreation, viewing, tourism, human activity, angling, fishing, photography, hiking, camping, aircraft, human conflict, human interaction, snow mobiling, snow machining, viewing (or bear-viewing), ATV, climbing, boating, rafting, kayaking, and hunting. All papers that pertained to bears and human recreational activities were included with no restrictions in the search on the year of publication. We summarized the findings from each study as to whether the activity caused spatial avoidance, temporal avoidance, changes in the time spent at a habitat, changes in the number of bears present, changes in sex/age class of bears in a habitat, or changes in activity budget. We also noted any results on the distance at which bears reacted to humans during an activity. A PRISMA diagram of the literature summarized is provided in S1 File.

### Survey of recreations and potential effects on brown bears

We elicited expert knowledge through a modified Delphi survey method [32, 33] on human recreational activities in bear habitat from 12 bear experts (hereafter, “Delphi Survey Experts”). Bear experts were identified by either 1) their scientific publications on the impacts of one or more human recreational activity on bears, or 2) their experience in managing bear populations impacted by human recreational activities. Experts were contacted initially either via phone or email and all experts that were contacted agreed to participate. The traditional Delphi method proceeds with additional rounds until the panel reaches consensus. We used a modified Delphi process to elicit individual contributions including individual expressions of uncertainty in the results [30]. Two advantages of using a modified Delphi survey method are the elimination of need for face-to-face contact and that the participants remain anonymous to each other, preventing domination by one or more individuals [31]. We selected a group of experts based on their collectively wide geographical experience with coastal North American (access to salmon), interior North American (limited or no access to salmon), and European brown bear populations. We considered European brown bears separately from North American brown bears as they have a longer history of co-existence with humans and occur in areas of higher human population density. In addition, European brown bears exhibit differences in behavior compared to North American brown bears as a result of being hunted to near extirpation over much of their European range during the last several hundred years [34, 35, 36]. European brown bears are also mainly nocturnal or crepuscular compared to North American brown bears, which are primarily crepuscular or diurnal in the absence of human disturbance [37, 38, 39, 40, 41]. Interior and coastal brown bears were separated based on access to salmon as the types and frequencies of recreational activities differ.

Twelve experts participated in the survey; three for European bear populations, six for interior North American bear populations, one for coastal North American bear populations, and three for both interior and coastal North American bear populations. We conducted the surveys via e-mail. We conducted several rounds of surveys structured and informed by the literature review. The first survey requested the Delphi Survey Experts to identify human recreational activities that occur in bear habitats that may impact bears, types of potential impacts these recreations might have on individual behavior and bear populations, potential benefits to bears resulting from those recreational activities, potential management actions to reduce negative impacts, and potential areas of research needed to fill key information gaps (S2 File). Bear hunting was not differentiated by the presence or absence of the use of bait or supplemental feeding. The second survey asked the Delphi Survey Experts to rank from Round 1 the recreational activities by greatest level of disturbance; the potential population level impacts by greatest impact; management actions by those most effective in minimizing impacts from recreational activities; and future research ([31, 42] S2 File). For the final round, the Delphi Survey Experts were asked to estimate the frequency of each recreational activity for the bear populations they have experience with, estimate the proportion of each specific bear population affected by each activity, and indicate the potential impacts of recreational activities in those bear populations (S2 File).

The frequency of occurrence of a recreational activity category and the proportion of the bear population affected are reported from the final survey as the mean and standard deviation of the ranks for each recreational activity for interior North America, coastal North America, and European brown bear populations. The management actions identified to minimize impacts of recreational activities are reported as a mean rank (with 1 being the activity most likely to reduce a given negative impact) and standard deviation. Results for the potential benefits to bears are reported as the percentage of experts who listed each item. Gaps in current knowledge are reported as those items ranked in the top third of results (a mean rank of less than 4). The highest priority for future research needs is ranked 1. We report the percentage of experts that noted potential for an impact on reduced survival, decreased nutritional intake, displacement, and reduced reproduction.

### Bayesian network model estimating potential impacts of human recreation on Alaska brown bears

**Fig 1:**
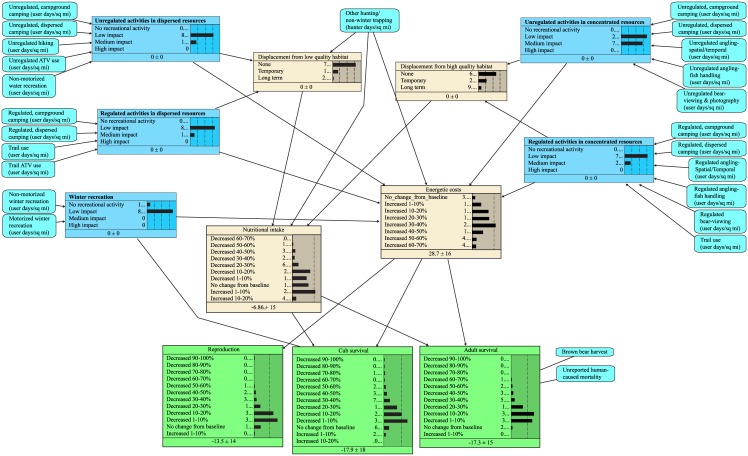
Bayesian network model examining the potential impacts of human recreational activities on Alaskan brown bears.

Using the knowledge gained from the Delphi Survey Experts, along with our literature review, a Recreation-Based Modeling Team structured the BNM (one of us also served as a Delphi Survey Expert) focusing on Alaskan brown bears, including coastal and interior populations. (Fig 1; Table A in S3 File). Initially, we intended to apply the Bayesian network model to brown bears throughout North America and Europe and therefore, we surveyed experts in these areas. Later the scope of the Bayesian network model was narrowed to include only Alaska brown bears due to perceived differences in bear behaviors, responses to recreation, and the types of recreations that are most common in Alaska versus other regions. The Modeling Team was composed of 5 experts with specific knowledge of Alaskan brown bears, drawn from state and federal natural resource management agencies in Alaska (S. Farley, K. Rode, G. Hilderbrand, C. Jorgensen, and J. Wilder). This sample size is considered appropriate in the development of ecological Bayesian network models [43] and similar sample sizes (e.g., 7) have been used in recent studies [44].

The BNM was developed as a tool for managers to evaluate the potential impacts of recreational activities on Alaskan brown bears in a manager’s local jurisdiction over a year’s time. Managers of Alaskan brown bears can run the model by determining the recreational activities that occur within their jurisdiction and selecting the user level for each input node in the model. The conceptual model and framework established here can be applied to other geographic areas and other species of bears by modifying the priors and conditional probability tables to reflect differences in behaviors and recreations.

#### Model structure

BNMs are influence diagrams that link variables, represented as nodes in the model, with probabilities. We assigned states to each node, and to each state we assigned unconditional (marginal) prior probabilities to input nodes or conditional probabilities (in conditional probability tables, CPTs) to all other nodes. We developed a BNM using the modeling shell Netica (vers. 4.16, Norsys, Inc., Vancouver, British Columbia) and guidelines from Cain [45], Marcot et al. [46, 47], and Conroy and Peterson [48], for developing modeling levels based on literature and expert knowledge, peer review, and model revision [47]. The Recreation-based Modeling Team (Table A in S3 File) populated the probability tables. Probabilities were informed from the expert knowledge of the Modeling Team who used established relationships in the literature and responses from the Delphi Survey Experts in their decision making.

Each recreational activity was depicted as the number of annual user days or user nights per square mile to accommodate variable jurisdictional sizes (Tables A and B in S3 File). We structured the BNM with five intermediate nodes to summarize recreational activities with similar impacts and to reduce model complexity (size of the conditional probability tables) [47]. Recreational activities, or inputs, are combined in summary nodes depending on whether the activities are regulated or unregulated and if they occur in habitat containing concentrated (e.g., salmon or salt-marsh meadows) or dispersed (e.g., moose or cow parsnip) food resources for bears. Berries may be considered as either a dispersed or concentrated food resource by managers depending on the concentration on the landscape, type of berry, and size and extent of the berry crop. Unregulated recreational activities include those that do not occur under management regulation or those for which regulations are unenforced. Winter recreational activities were grouped into a separate summary node because they impact bears during the denning season.

The nodes representing brown bear harvest and other hunting and non-winter trapping were not included in summary nodes of other recreational activities because their impacts differ from most other recreational activities. These activities were not combined into an intermediate “hunting” node because their individual effects differ. Other hunting may increase nutritional intake when bears feed on big game carcasses [49, 50]. Bear hunting also directly impacts survival whereas other hunting leads more to disturbance and has the potential for defense of life and property kills. Bear hunting and other hunting input nodes include subsistence and sport hunting because subsistence and sport hunting often occur simultaneously and the biological impacts on bears cannot be differentiated.

The Recreation-Bear Modeling Team developed an index of impact on bears for each recreational activity using group consensus. The Recreation-Bear Modeling Team assigned a value of 0–5 (0 = not applicable, 1 = lowest impact, and 5 = highest impact) to each recreational activity and state combination based on the relative impact compared to other recreational activities (Table C in S3 File). Recreational activity states of ‘none’ were assigned an index value of zero. Model summary nodes are discrete and their conditional probability tables are based on simple additive influences of their recreation-activity input nodes as a parsimonious (linear) expression of how recreational activities compound. The model might underestimate adverse impacts if higher-order non-linear combinations of recreational activities occur. The states for the summary nodes were specified to be no recreational activity, low impact, medium impact, or high impact. Impact levels were calculated, not assigned, at the summary nodes by calculating the possible points for that node (i.e. the product of the number of recreational activities x 5; the highest impact score assigned). Next, the maximum number of points was divided into thirds. The lowest third was assigned to low impact, the medium third to medium impact, and the highest third to high impact (Table D in S3 File). Although it is not possible for the following states to occur under the current index assignments, they were maintained in the model to allow for future modifications of the model should data become available to refine the model: 1) medium impact for winter recreation; and 2) high impact for winter recreation, unregulated activities in dispersed resources, regulated activities in dispersed resources, and regulated activities in concentrated resources.

The mechanisms of impact (represented by the intermediate nodes in the model) included displacement from high and low quality habitat, energetic costs, and nutritional intake. These mechanisms were based on the results from the Delphi Survey Experts and the literature review. Further, these states were considered separately to aid experts in associating the specific mechanisms by which bears may be affected by various recreations. We defined high quality habitats as those that contain concentrated food resources (e.g., salmon streams or salt-marsh meadows) and low quality habitats as those containing dispersed food resources (e.g., moose or cow parsnip). Displacement of bears was defined as bears leaving the immediate area to avoid humans either temporally or spatially. Long-term displacement was defined when bears were displaced from the resource during the entire time it was available that year. The intermediate nodes of displacement from low and high quality habitat are discrete and measured by the probability of being displaced given the type and intensity of recreational activities. User inputs into the models account for seasonal variability in resources by indicating the degree to which activities occur in habitats of high or low quality. For example, if human recreation occurs on a salmon stream during a period when bears don’t typically fish, a user’s input would reflect that as an activity occurring in a low quality, rather than high quality habitat.

Output nodes are cub survival, reproduction, and adult survival. The intermediate nodes of nutritional intake and energetic costs and the three output nodes are all continuous variables, depicting percent increase or decrease over baseline (i.e. normal background levels). We discretized the states of these nodes into 10% categories based on our experts’ advice. Each expert individually assigned a percent increase or decrease for each individual node state in a CPT for the intermediate and output nodes. The CPTs were then populated by treating the individual input node states as additive. Probabilities were assigned to each category based on the percentage of the Recreation-Bear Modeling Team whom assigned a value within that category (Table E in S3 File). The Modeling Team considered whether effects on reproduction and survival estimated in the model were within known variabilities of those metrics in the wild.

#### Model limitations and assumptions

The killing of a bear in defense of life and property (DLP) is defined in Alaska as occurring if a person did not provoke the attack or cause a problem by leaving attractants in a manner that attracts bears and if the person had done everything else possible to protect their life and property [51]. Initially, we included DLPs in the model as a mechanism of the impact of recreational activities. However, we reviewed the available records for reported DLPs from 1987–2012 for incidences that involved recreational activities and determined that the occurrence in Alaska is extremely low (Alaska Department of Fish & Game, unpublished data), so we did not include DLPs in the model. There are approximately 13 reported DLPs per year across the state of Alaska that occur as a result of recreational activities; hunting for game other than bears accounted for 78% of these incidences. When compared to the hundreds of thousands of recreational users across the state in a given year, the probability of a DLP is less than one percent for other hunting and even lower for all other recreational activities.

Assumptions in creating the model included the following. 1) There is a spectrum of bear behavior dependent on the history of interactions with humans that ranges from urban (e.g., Anchorage) to naïve (e.g., remote wilderness) populations. We designed the model for bear populations in the middle of that spectrum, i.e., for bears that live in wild (non-urban) areas with a history or current instances of human visitation. 2) For regulated recreational activities represented in the model, we assumed a high compliance to management regulations or guidelines that minimize bear-human conflicts from users regardless of mechanisms for enforcement. If regulations or guidelines are unenforced, then the recreational activity should be specified in the model as “unregulated.”

The output node of adult mortality included male and female bears. Although adult female mortality is the driving force of population dynamics [52, 53], the BNM is not intended to be a population dynamics model. Also, the impacts of recreational activities occur at the individual level and affect males and females, which is important to track for local management. However, reproduction, cub survival, and adult survival are all demographics that influence population stability and are used to calculate population trends [54].

The prior probabilities for all recreational activities, that are the input nodes in the model, were set to uniform distributions by which to represent equal uncertainty across conditions before a situation is specified when running the model.

S4 File contains a user manual to guide managers in their application of the BNM.

#### Model verification

The model was initially structured and probability values were assigned to create an “alpha-level” model (S3 File, sensu [55]), which was then provided to an outside subject-matter expert (D. Gustine, U.S. Geological Survey) for peer review. The peer reviewer assessed the model’s structure and probability values with the modeler (J. Fortin-Noreus) to suggest edits or to confirm the model’s construction. The BNM panel of five experts then evaluated the peer review and suggested revisions to the model as necessary to create a beta-level model (S3 File). Finally, we performed sensitivity analyses to determine which recreational activities have the most significant effect on outcomes and to prioritize future research by identifying recreational activities that could most affect bear disturbance and that might be poorly studied or least understood. For the sensitivity analysis, prior probabilities of the input nodes were set to their default uniform distributions. We also conducted influence runs to compare all possible states of the nodes brown bear harvest and unreported human-caused mortality, with all other input nodes set to their default uniform distributions. Influence runs are conducted by setting the selected sets of input variables to their most positive or negative states and comparing the resulting model outcomes [56, 57, 58].

We ran the model under five controlled scenarios and three management scenarios. The controlled scenarios were: 1) recreational activities are unregulated and resources are dispersed; 2) recreational activities are regulated and resources are dispersed; 3) recreational activities are unregulated and resources are concentrated; 4) recreational activities are regulated and resources are concentrated; and 5) all recreational activities occurring simultaneously in areas of both concentrated and dispersed resources. For each scenario the selected recreational activities were assigned a state of high or common to compare the maximum impacts to brown bears in the BNM (Table F in S3 File). The three management examples include: 1) regulated, high visitor use focused on bear-viewing and angling/no hunting: Brooks Camp in Katmai National Park, Alaska; 2) unregulated, low visitor use focused on bear-viewing: Hallo Bay in Katmai National Park, Alaska/no hunting; and 3) unregulated and regulated, very high visitor use primarily focused on angling with hunting for other species: the Kenai-Russian River Management Area on the Kenai Peninsula, Alaska (i.e., Russian River; Table G in S3 File). Visitors to Brooks Camp and Hallo Bay in Katmai National Park are there primarily for bear-viewing and angling. However, visitor impact differs between the areas. Brooks Camp receives high numbers of visitors, and their activities are regulated, whereas Hallo Bay has far fewer visitors and unregulated activities. Brooks Camp also has a high level of regulated campground camping. The Russian River has the highest visitor use of the 3 scenarios with participation in both unregulated and regulated activities as a result of the river banks being managed by different agencies with different sets of regulations. Most visitors participate in angling, in addition to high levels of campground camping, and opportunistic bear-viewing. At the Russian River, visitor levels are distributed between regulated and unregulated activities for angling and campground camping based on compliance monitoring (Bobbie Jo Skibo, pers. comm.). The Russian River also is an area where hunting for species other than bears occurs whereas both scenario 1 and 2, Brooks camp and Hallo Bay, are within Katmai National Park where hunting does not occur.

## Results

### Literature review

We identified 46 articles ranging in publication date from 1972 to 2013 containing original data on recreation impacts on brown bears. Potentially negative impacts on brown bears were reported for bear-viewing (18 publications), hiking (11), angling (10), camping (4), bear hunting (3), ungulate hunting (3), non-motorized winter recreation (3), and 1 each for motorized winter recreation, mountain climbing, ATV use, and motorized watercraft (sum >46 because some articles addressed >1 category). Spatial and temporal avoidance (“displacement” in our BNM) was cited as the most common response to recreational activities with angling and bear-viewing being the most frequently studied recreational activities.

Spatial avoidance includes bears avoiding areas close to humans and leaving areas in response to humans, either when humans arrive or when humans approach within a specific distance. Bears’ avoidance of areas close to humans is often measured by defined zones during scan sampling or by analyzing habitat use identified by recording locations of bears. Bears commonly avoid the same areas of streams used by anglers [59, 60, 61], bear-viewers [62, 63, 64] and hikers [60, 65]. On salt marshes, bears avoid foraging within 600m of bear-viewers [63]. Habitat use by bears was less than expected near non-motorized trails [66, 67, 68], ATV trails [69] and campsites [67, 70, 71]. Bears fled the area in response to motorized watercraft [72], mountain climbers [73], trail hiking [74, 75], and off-trail hiking [74].

The distance at which bears walked or ran from humans on foot varied with recreational activity, location, and type of approach. Most coastal brown bears walked or ran away from bear-viewers and anglers when less than 100m away [72], although bears were less likely to flee during years of controlled bear-viewing, areas where there are spatial and temporal regulations on bear-viewing. Bears that were directly approached by hikers fled at distances from 100 to 400m, whereas bears that were more tangentially passed by hikers tolerated distances <100m [76, 77, 78, 79, 80]. Bears that were passed by hikers fled at longer distances when the bears were active than when they were inactive when they first encountered people [79]. Bears in open habitats fled from humans at greater distances than did bears in closed habitats [81]. In areas where hiking occurred, bears increased their use of covered habitats [70, 76].

Temporal avoidance was defined in the literature as bears changing the time of day that they are active in response to human presence. Brown bears switched from diurnal to crepuscular or nocturnal activity in response to bear-viewing [63, 82, 83, 84], angling [60, 83], hiking [68, 80], camping [85], and bear hunting [80]. Males were less active during the day when bear-viewers were present if humans acted predictably, compared to their activity patterns when bear-viewers were absent [86, 87]. Whereas females with cubs were more active when bear-viewers were present, no change was observed for subadults and lone adult females [86]. For brown bears feeding on salmon and berries, lone adults and family groups were more night active in high human use areas compared to low human use areas, in contrast to subadults, which were more day active in both areas [39].

Bears were present in decreased numbers and/or for shorter periods of time when exposed to people angling [59, 88, 89], bear-viewing [39, 62, 63, 64, 89, 90, 91], and mountain climbing [73]. Fewer bears were present at coastal foraging and salmon feeding sites when bear-viewers were present compared to when bear-viewers were absent [64]. Bears decreased their length of stay at streams in areas with angling [59, 88, 89]. The number of single adults and family groups increased during years of controlled public access when compared to years of uncontrolled public access [62, 90, 91]. When viewers were present 24-hours a day, bears spent less time on a salmon stream when compared to daytime-only viewing [63].

Bears spent less time fishing when anglers and bear-viewers were present [83] and had decreased fishing success [83, 92] compared to when anglers and bear-viewers were absent. In areas where males were displaced by bear-viewing [87, 93] or angling and bear-viewing [92], an increase in females with cubs was sometimes seen. In areas where adult male brown bears temporally avoid bear-viewers and anglers, females with cubs have increased access to salmon [59, 61, 63, 75, 84, 86, 87, 91, 92, 93, 94].

Bear hunting can lead to increased wariness [95, 96], increased use of cover [97], increased nocturnal and decreased diurnal activity [98], and increased reaction distances to human activities [99]. However, in areas with big-game hunting for species other than bears, an increase in bear presence may occur as they feed on carcass remains [49, 50]. As a result, bears may become food-conditioned, resulting in increased human-caused bear mortalities (DLPs and management removals) and an increased risk of injuries to humans [29, 50, 100, 101].

Brown bears are infrequently approached by researchers during denning, but when they are, den abandonment may occur [102, 103, 104]. We assumed that reactions to the approach of researchers to dens reflected reactions similar to those that might occur in response to recreational hiking, snow shoeing, or skiing. Den abandonment sometimes resulted in the abandonment of cubs and resultant cub mortality in black bears [105, 106]. Although cub abandonment has not been documented in brown bears, females that abandoned a den prior to parnutrition were more likely to lose young in the den over the winter [104]. Motorized winter recreational activities can also cause den abandonment [104].

### Survey of recreations and potential effects on brown bears

**Fig 2:**
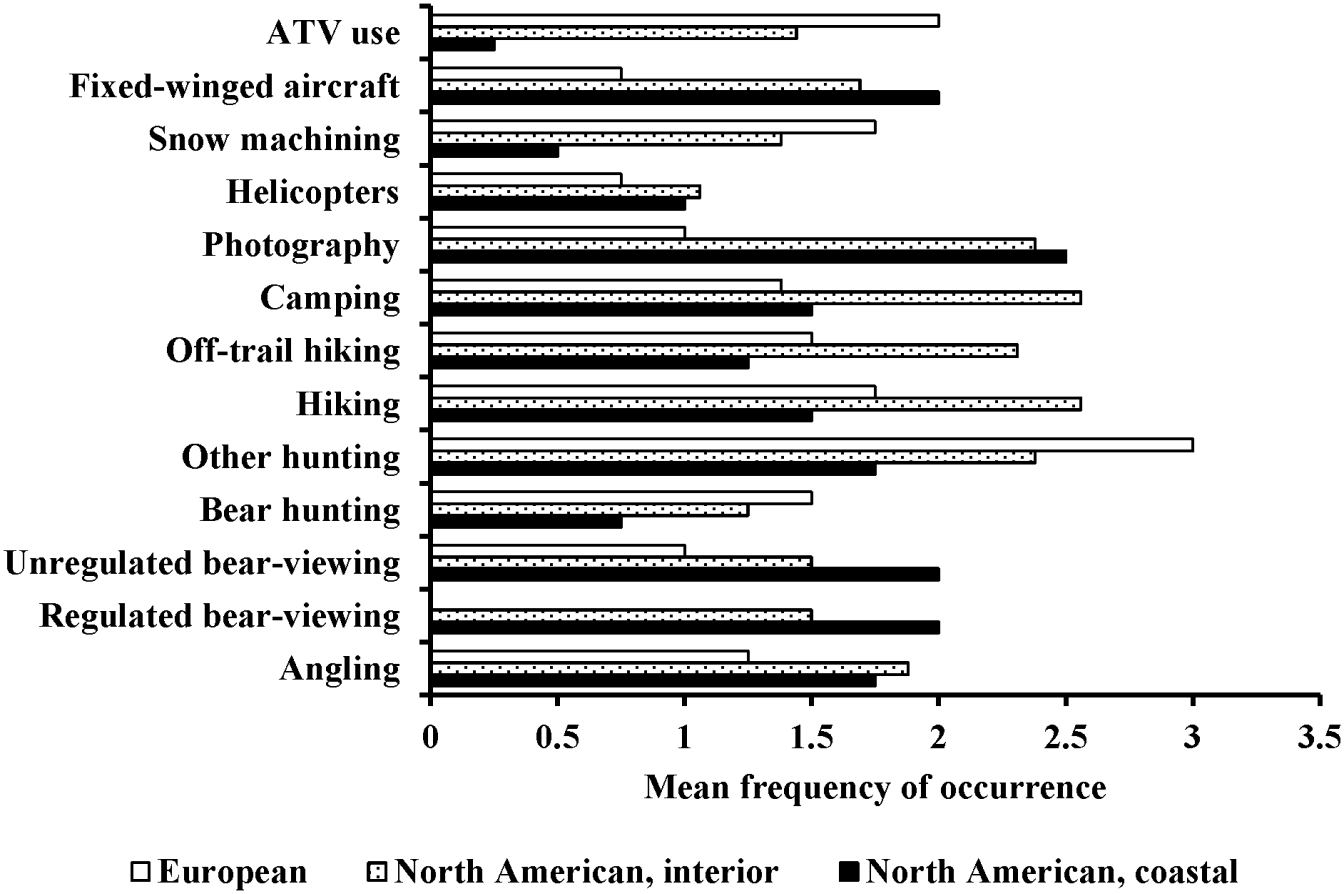
Occurrence of human recreations in habitats of coastal and interior brown bears in North America and European brown bears (0: Does not occur, 1: Rare, 2: Common, 3: Very common) by 12 experts in a modified Delphi survey.

**Fig 3:**
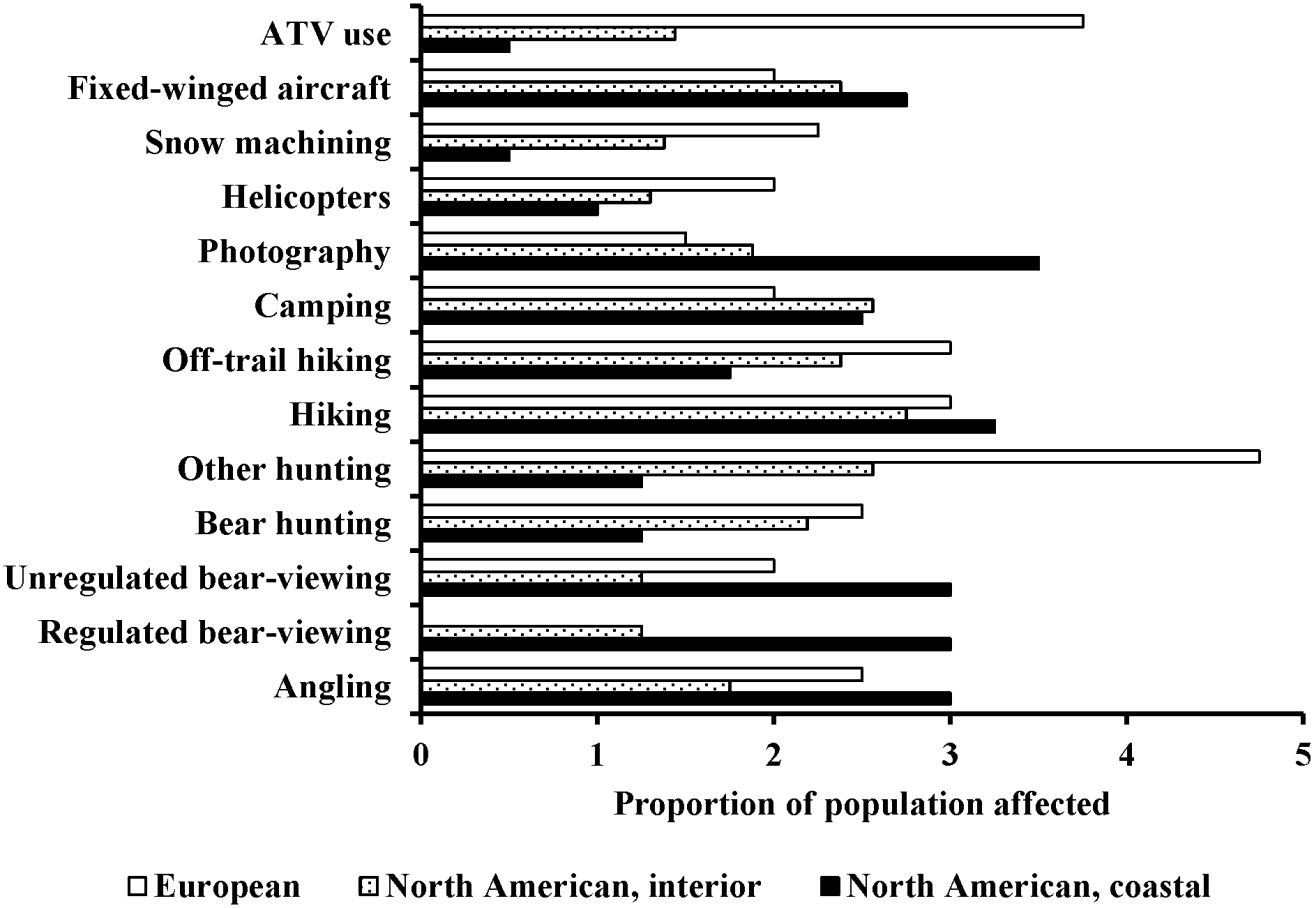
Expert ranking of the proportion of coastal and interior brown bear populations in North America and European brown bear populations affected by human recreational activities (Does not occur, 1: 0–5%, 2: 5–35%, 3: 35–65%, 4: 65–95%, 5: 95–100%) provided by the 12 Delphi Survey Experts.

We separated the frequency of recreational activities, and the proportion of brown bear populations affected, by the geographic areas of (1) coastal North American (access to salmon), (2) interior North American (limited or no access to salmon), and (3) European. We based those separations on differences in types of recreational activities, behavioral responses, and population dynamics as scored by the 12 Delphi Survey Experts (Figs 2 and 3). The Delphi Survey Experts identified the top five most frequent recreational activities in habitats of coastal North American bears as photography, regulated bear-viewing, unregulated bear-viewing, fixed-winged aircraft use, and angling. All of these activities, plus hiking, were ranked by the 12 bear experts as affecting >35% of a given population. In habitats of interior North American bears, the most frequent recreational activities identified by the 12 bear experts were trail hiking, camping, other hunting, photography, and off-trail hiking. All of these activities were ranked by the bear experts as impacting <35% of a given population. The most frequent activities in European brown bear habitats were identified as: other hunting, ATV use, snow machining, hiking, bear hunting, and off-trail hiking. Of these, other hunting, hiking, off-trail hiking, and ATV use were ranked as impacting ≥35% of the population for a given instance of any one of these activities.

The Delphi Survey Experts were most frequently concerned with displacement of individuals in a given population impacted by recreational activities (Table 1). More than 50% of experts from all geographic regions believe that hunting for game other than bears has the potential to reduce individual bear survival. Decreased nutritional intake was identified as a response to angling, regulated bear-viewing, unregulated bear-viewing, and photography for coastal North American bears. Reduced survival was identified for some activities as a consequence of defensive kills, but reduced reproduction was not an impact of major concern for any of the populations regardless of type of recreational activities.

**Table 1:** The percentage of 12 experts participating in a modified Delphi survey that attributed the potential for impact (RS: reduced survival; DNI: decreased nutritional intake; D: displacement; RR: reduced reproduction) on coastal and interior black and brown bears in North America and European brown bears for each recreation if left unmitigated.

Human recreational activity	Coastal bears				Interior bears				European bears			
	RS	DNI	D	RR	RS	DNI	D	RR	RS	DNI	D	RR
Angling	25	100	100	25	13	50	50	13	0	25	50	0
Regulated bear-viewing	25	75	75	25	0	0	25	0	0	0	0	0
Unregulated bear-viewing	25	100	100	25	13	38	50	0	0	50	75	0
Bear hunting	100	50	75	50	63	25	50	50	50	25	25	0
Other hunting	75	50	100	25	63	25	50	38	100	50	75	0
Hiking	25	25	100	25	13	38	75	0	0	25	100	0
Off-trail hiking	25	25	100	25	13	13	63	0	0	25	100	0
Camping	25	50	75	25	50	25	75	0	0	0	50	0
Photography	25	75	75	25	13	25	50	0	0	0	50	0
Helicopters	25	25	50	25	0	13	38	0	0	0	0	0
Snow machining	25	25	50	25	25	13	38	25	0	0	50	25
Fixed-winged aircraft	25	50	50	25	0	25	38	0	0	0	0	0
ATV use	25	25	50	25	13	13	38	13	50	25	75	0

The Delphi Survey Experts ranked the potential effectiveness of management actions to reduce the impacts from recreational activities on bears (Table A in S2 File). The management actions in order of mean rank among the experts (with a rank of 1 being the most effective) were: education of the public (2.8); control of food and garbage (3.2); regulate road density (3.6); manage hunting regulations and educate hunters (4.3); create and reinforce existing protected areas (4.7); seasonal closures of high density bear areas (5.3); campground and trail placement (6.0); regulate bear-viewing (6.1); regulate angling (6.2); and temporally control access (7.5). The Delphi Survey Experts’ reasons behind their ranking of the management actions are given in Table A in S2 File.

The potential benefits to bears as a result of recreational activities were (presented by the of Delphi Survey Experts): 1) an increase in conservation or support for bears and habitat through an improved understanding and appreciation of bears (62%); 2) economic benefits with an increase in revenue for local economies (23%); 3) access to prime habitat by females with cubs and subadults where dominant bears avoid humans (23%); 4) areas used for bear-viewing may be protected from bear hunting (15%); 5) enhancement of fish populations for recreational angling may increase the food supply for bears (8%); and 6) the construction of hiking and biking trails open up easy travel paths for bears (8%). 23% of respondents indicated that recreational activities had no benefit to bears.

The Delphi Survey Experts identified many gaps in current knowledge regarding the impacts of recreational activities on bears (Table B in S2 File). The most critical gaps in current knowledge (mean assignment, with 1 being the most important out of a possible 10) were: 1) identify what the relationship is between habituation, displacement, and stress caused by recreational activities (2.8); 2) people’s attitudes towards bears and people’s tolerance levels (3.2); 3) displacement from salmon streams by anglers (3.4); 4) identify prime habitats for human exclusion (3.6); 5) evaluate population impacts from recreational activities (3.7); and 6) identify and quantify the number of people participating in recreational activities in bear habitat (3.9).

### Bayesian network model estimating potential impacts of human recreation on Alaska brown bears

The primary drivers of model outcomes were the presence or absence of regulation of recreational activities and if the activities occurred where resources for bears were dispersed or concentrated. The probabilities of temporary and long-term displacement were 0.9 and 2.7 times greater, respectively, from high quality compared to low quality habitats (Table 2). The probabilities of displacement from both low and high quality habitats were higher with unregulated activities than with regulated activities (Table 2). When the probability of displacement is equal from low and high quality habitats, the decrease in nutritional intake was greater from high quality habitats compared to low quality habitats. All output and intermediate node outcomes for unregulated compared to regulated recreational activities were greater in concentrated compared to dispersed bear resources (Table 3). The percent decrease in nutritional intake was up to 1.9 times greater, the percent increase in energetic costs was 3.4 times greater, and reproduction, cub survival, and adult survival decrease up to 2.2, 2.1, and 2.0 times more, respectively, when unregulated recreational activities are compared to regulated recreational activities. When recreational activities occurred in areas containing concentrated resources compared to dispersed resources, the percent decrease in nutritional intake was up to 5.3 times greater, the percent increase in energetic costs was 2.1 times greater, and reproduction, cub survival, and adult survival decreased up to 3.0, 2.8, and 2.6 times more, respectively.

**Table 2:** Probabilities of temporary or long-term displacement (i.e., for the duration of seasonal or annual use of a habitat) under various Bayesian network model management and environmental scenarios for Alaskan brown bears including: 1) unregulated recreations in bear habitat with dispersed resources; 2) regulated recreations in bear habitat with dispersed resources; 3) unregulated recreations in bear habitat with concentrated resources; 4) regulated recreations in bear habitats with concentrated resources; and 5) all recreational activities in either low or high quality habitats. Low quality habitats are those in which resources are dispersed. Therefore, only scenarios 1, 2, and 5 result in probability outcomes. High quality habitats are those in which resources are concentrated. Therefore only scenarios 3, 4, and 5 result in probability outcomes.

	Probability of displacement			
	Low quality habitat		High quality habitat	
Scenario	Temporary	Long-term	Temporary	Long-term
1. Unregulated recreations in dispersed resources	12	2	–	–
2. Regulated recreations in dispersed resources	10	3	–	–
3. Unregulated recreations in concentrated resources	–	–	40	29
4. Regulated recreations in concentrated resources	–	–	22	7
5. Both unregulated & regulated recreations in both dispersed & concentrated resources	33	7	56	19

**Table 3:** The expected percent increase or decrease in nutritional intake, energetic costs, cub survival, and adult survival of Alaskan brown bears under the following recreation scenarios. Results are probability outcomes (± 1 standard deviation in probability) in the Bayesian network model relative to outcomes if there was no recreational activity: 1) unregulated recreation in bear habitats with dispersed resources; 2) regulated recreation in bear habitats with dispersed resources; 3) unregulated recreation in bear habitats with concentrated resources; 4) regulated recreation in bear habitat with concentrated resources; and 5) all recreational activities occurring.

	Percent change in probability relative to no recreation				
	Nutritional intake	Energetic costs	Reproduction	Cub survival	Adult survival
1. Unregulated recreation in dispersed resources	-2 ± 5	9 ± 6	-5 ± 5	-6 ± 6	-5 ± 5
2. Regulated recreation in dispersed resources	-2 ± 5	6 ± 6	-4 ± 5	-5 ± 6	-4 ± 5
3. Unregulated recreation in concentrated resources	-11 ± 1	27 ± 15	-14 ± 16	-19 ± 19	-12 ± 12
4. Regulated recreation in concentrated resources	-7 ± 12	8 ± 7	-7 ± 10	-9 ± 11	-6 ± 7
5. Both unregulated & regulated recreations in both dispersed & concentrated resources	-13 ± 18	49 ± 17	-22 ± 20	-30 ± 25	-25 ± 20

Among the three management scenarios examined, probabilities of displacement were slightly higher for temporary and long-term displacement from high quality habitat where high levels of regulated use occurred (scenario 1: Brooks Camp, Katmai National Park, Alaska) compared to low levels of unregulated use (scenario 2: Hallo Bay, Katmai National Park, Alaska). This lead to greater declines in nutritional intake, reproduction, cub survival, and adult survival with high levels of regulated use compared to low levels of unregulated use (Tables 4 and 5). Where high levels of regulated and unregulated angling and camping occurred (scenario 3: Russian River, Alaska) the probability of temporary displacement from high quality habitat was similar to areas of regulated, high visitor use bear-viewing and angling (scenario 1) but the probability of long-term displacement was higher (Table 4). Although the change in nutritional intake for scenario 3 was in between that of the other two scenarios, the increase in energetic costs was double that of the other two scenarios. This translated into a greater reduction in reproduction, cub survival, and adult survival in this scenario compared to the other 2 (Table 5). The much higher energetic costs in scenario 3 was a function of higher levels of regulated and unregulated activities overall and the presence of other hunting.

**Table 4:** Comparison of expected probabilities of displacement, temporary and long-term (i.e., for the duration of seasonal or annual use of a habitat) of Alaskan brown bears from low (i.e., dispersed resources) or high quality habitat (concentrated resources) under the following recreation scenarios based on the Bayesian network model outcomes. 1) regulated, high visitor use bear-viewing and angling; e.g., Brooks Camp in Katmai National Park and Preserve, Alaska; 2) unregulated, low visitor use bear-viewing; e.g., Hallo Bay in Katmai National Park and Preserve, Alaska; and 3) regulated and unregulated angling and camping; e.g., the Kenai-Russian River Management Area, Alaska.

	Probability of displacement			
	Low quality habitat		High quality habitat	
Scenario	Temporary	Long-term	Temporary	Long-term
1. Regulated, high visitor use; primarily viewing	6	1	29	7
2. Unregulated, low visitor use; primarily viewing	6	1	22	2
3. Regulated and unregulated high visitor use; primarily angling and camping	10	1	29	11

**Table 5:** The expected percent increase or decrease in nutritional intake, energetic costs, cub survival, and adult survival of Alaskan brown bears under the following scenarios. Results are probability outcomes (± 1 standard deviation in probability) in the Bayesian network model relative to outcomes if there was no recreational activity: 1) regulated, high visitor use, primarily bear-viewing and angling; e.g., Brooks Camp in Katmai National Park and Preserve, Alaska; 2) unregulated, low visitor use, primarily bear-viewing; e.g., Hallo Bay in Katmai National Park and Preserve, Alaska; and 3) regulated and unregulated, high visitor use, primarily angling and camping; e.g., the Kenai-Russian River Management Area, Alaska.

	Percent change in probability relative to no recreation				
	Nutritional intake	Energetic costs	Reproduction	Cub survival	Adult survival
1. Regulated, high visitor use, primarily viewing & angling	-7 ± 12	12 ± 9	-8 ± 11	-12 ± 12	-7 ± 8
2. Unregulated, low visitor use, primarily viewing	-6 ± 11	12 ± 9	-7 ± 10	-9 ± 11	-6 ± 7
3. Regulated & unregulated, high visitor use, primarily angling & camping	-7 ± 13	25 ± 13	-13 ± 13	-16 ± 15	-12 ± 12

Sensitivity analysis, based on all activities set to the default of uniform distribution, showed that the BNM outputs of reproduction and cub survival were most sensitive to unregulated recreational activities in areas where concentrated resources occur (Table H in S3 File). Nutritional intake had a greater impact on cub survival and reproduction than energetic costs. Given equal changes in nutritional intake and energetic costs, cub survival was impacted more than reproduction. Adult survival was most sensitive to unregulated activities in concentrated resources in the absence of direct human-caused mortality. However, an influence run showed that brown bear harvest and unreported human-caused mortality were overwhelmingly influential to adult survival at any level other than none and not influential, respectively. Given the difference between direct and indirect mortality, we anticipated this result.

## Discussion

The literature review and the Delphi Survey Experts identified spatial and temporal displacement as the most common impact of human recreational activities on bears. Most studies have focused on behavioral responses and the common recreational activities of bear-viewing and angling. Empirical studies and expert knowledge suggested that recreational activities secondarily affect bears through reduced food intake, either as a result of displacement or a change in time spent feeding, and less frequently through changes in the sex and/or age composition of bears at food resources. The BNM results further suggest that increased energetic costs associated with displacement may be a primary mechanism by which recreation affects bear health with consequent population-level effects.

Displacement may be reduced in areas where bear-viewers or anglers behave predictably [62, 63, 72, 90, 91, 92]. Predictable recreational activities allow individual bears to either habituate to the presence of humans [90, 107, 108], temporally avoid humans [86, 92], or spatially avoid humans [109], thereby reducing bear-human interactions [110, 111]. Predictable recreational activities can be spatially controlled, e.g. bear-viewing from designated platforms, or temporally controlled, by limiting bear-viewing hours, to allow bears to access the resource while avoiding humans. Temporally displaced bears at salmon streams may not experience a decreased fishing rate because darkness may reduce the evasive responsiveness of salmon and salmon are more active at night [93, 112]. The BNM gives managers the ability to compare changes in nutritional intake, energetic costs, reproduction, cub survival, and adult survival as they make changes in the model to the levels of recreational activities and whether the activities occur in a regulated or unregulated manner.

Recreational activities may alter the sex and age classes that use habitats and food resources when males are the primary group displaced. Dominant adult males fish the most productive stream areas [113] while females with cubs may avoid large males to reduce the risk of infanticide [114, 115, 116]. Although subadults and lone adult females may also be at risk of intraspecific aggression [113, 117], they do not always avoid large males to the degree that females with cubs do [115]. Nevin and Gilbert [118] concluded that a positive effect of ecotourism is increased access to salmon by females as female reproductive success is positively correlated to meat intake and mean female mass [119]. However, other studies suggest that the presence of large males is a reflection of salmon or other food availability rather than the presence of bear-viewers [84, 90, 117, 120, 121].

Decreased caloric intake may occur if bears spend less time fishing [59, 88, 89] or foraging [84] as a result of human presence. In most studies however, the effect of decreased foraging on total food intake and individual health were not measured. In one study, spatially and temporally predictable bear-viewing and simulated angling were introduced and resulted in minimal effects on total food intake at salt marshes and salmon streams, with the exception of large males at salt marshes [84]. However, effects on reproduction and survival have never been confirmed for any recreational activity in studies to date.

By incorporating expert knowledge this study can begin to reduce uncertainty about the potential effects of recreational activities on individual health, reproduction, and survival to inform management. The Delphi Survey Experts suggested that a larger proportion of coastal bears compared with interior bears are affected by recreational activities resulting in displacement and decreased nutritional intake. As seen in the BNM outcomes, this may reflect a greater impact when displaced from concentrated resources, such as salmon streams. However, reduced reproduction was not a significant concern of the Delphi Survey Experts and the BNM, even with all recreational activity inputs set to the highest levels, reproduction decreased only 22 ± 20%. Cub survival decreased more (30 ± 25%), because decreased nutritional intake in adult females initially affects cub size and survival and only affects reproduction once adult female body condition is very poor [54, 122, 123].

Adult survival decreases the most when brown bear harvest is managed for population reduction, unreported human-caused mortality is set to influential, and all other recreational activities are set at the highest levels (36 ± 21%). In comparison, if neither brown bear harvest nor unreported human-caused mortality is present then the effect on adult survival declines to 19 ± 18%. It is not surprising that direct mortality caused by hunting and other human-caused mortality has such a large impact compared to the indirect effects of decreased nutritional intake and increased energetic costs. However, many managers may have more influence over how recreational activities occur (i.e. regulated versus unregulated) and at what levels recreational activities occur. Access to food and garbage is a primary cause of bear-human conflicts and of management removals in North America [24, 28, 124, 125]. Although management removals and defense of life and property killings were not mechanisms incorporated into the BNM (based on data suggesting very low levels of DLPs associated with recreation in Alaska), regulation of recreational activities in concentrated resources can cut the decline in adult survival in half by mitigating effects on nutritional intake and energetic costs associated with displacement. Managers can also reduce by half the adverse effects on cub survival, reproduction, and adult survival by reducing the levels of recreational activities from high to medium (e.g. decrease the annual number of bear-viewers from ≥10 to 3–10 per square mile).

### Access

Although presence of roads and human developments are not recreational activities, they were identified by the Delphi Survey Experts as having a significant impact on bears. The concern was greatest for interior and European bears with 88% and 100% of experts, respectively, ranking roads in their top five human recreational activities impacting bears. Roads are a significant source of direct human-caused bear mortality and increase access for human recreational activities, which in turn increases the potential for bear-human conflicts [126]. Increased access may also lead to an increase in direct mortality, through hunting and poaching [126, 127]. Both brown and black bears have been shown to spatially or temporally avoid roads and park developments [66, 99, 127, 128, 129, 130, 131]. For coastal brown bears only 38% of experts ranked roads in the top five human recreational activities impacting bears, probably because of the lack of roads in Alaska where the primary means of access is fixed-wing aircraft, boats, and helicopters. Helicopters and fixed-wing aircraft may lead to displacement and changes in habitat use [72, 81, 132]. Given the link between access and levels of human recreational activities, roads, helicopters, and fixed-wing aircraft need to be studied for direct and indirect impacts on bears.

### Management Implications

Education of the public was listed by the Delphi Survey Experts as the most effective management action in minimizing the impacts of recreational activities on bears. Information and education on the impacts of careless, unskilled, and uninformed actions are much more effective than regulations in changing the behavior of outdoor recreationists [133]. Most defensive attacks result from surprise encounters involving humans hiking off-trail, in the backcountry, and in areas of natural food abundance for grizzly bears [34, 124]. Education on how to respond during a bear encounter, proper use of bear deterrents (i.e. bear-spray), and where bears are likely to occur based on natural food availability could help reduce human-bear conflicts and adverse outcomes of encounters.

Proper storage of food and garbage to minimize bear-human conflicts was the second most effective management action identified by the Delphi Survey Experts. Improper storage of food and garbage is a primary cause of human-bear conflicts in North America [24, 25, 34, 124, 125]. The Recreation-Based Modeling Team identified a significant difference in the level of impacts a recreational activity has on bears based on proper storage of food, garbage, and caught fish. They therefore defined regulated versus unregulated camping as being with or without proper food and garbage storage and regulated versus unregulated angling as whether proper cleaning and handling of fish did or did not occur. Although we did not include DLPs in the BNM because of the low level of reported incidences in Alaska, this may need to be reconsidered for future models if bear-human DLP interactions increase, possibly as a result of location (i.e. wild-urban interfaces) or an increase in the level of recreationists.

Multiple management actions were identified by the Delphi Survey Experts and literature review that can reduce displacement and the potential for human-bear interactions. One such management action is to identify and protect, through permanent, seasonal, or daily closures, prime bear habitat for feeding and travel corridors. The placement of campgrounds, trails, and bear-viewing sites outside of prime bear habitat can reduce potential bear-human interactions and impacts on bears [95, 134, 135]. Campgrounds located within habitats containing natural food items have led to an increase in incidences of bear-human conflicts [136, 137]. The BNM structure reflects the larger impact recreational activities have when they occur in high quality habitat compared to low quality habitat because of the concentrated food resources they contain.

### Future Research

The Delphi Survey Experts identified as a top research priority the interaction between habituation, displacement, and stress resulting from recreational activities. Although some research has implied that bears habituate in response to recreational activities, habituation is difficult to measure and it is unknown what proportion of individuals habituate and under what specific conditions it occurs. Habituation may lead to increased opportunities for bear-viewing without displacing bears [120, 138] and decreased bear-human conflicts [78, 90, 108, 136]. However, some evidence supports an increased risk of bear-human conflicts in areas where bears are habituated [25, 34].

Identification of people’s attitudes towards bears and tolerance levels was also ranked highly as an information need by the Delphi Survey Experts. Understanding the underlying beliefs of human attitudes enables those beliefs to be changed, if necessary, through education and for support to be gained when implementing change [139]. People have more positive attitudes with increased knowledge about carnivores [140].

Other priority research objectives relate to studying long-term individual or population level effects of recreational activities because most research to date has evaluated only immediate, individual behavioral responses. Identification of prime bear habitat would also allow for the creation of seasonal, temporal, or zone closures to minimize the impacts on bears.

Controlled studies, such as those that include observations when recreationists are absent, will improve our knowledge of behavioral responses and how they vary by sex and age class and reproductive status. Only a handful of studies have done 24-hr observations to determine what bears are doing when humans are not present [63, 92]. Multi-year studies are important because the availability of food resources varies annually and may be an important interactive factor determining the effect of recreation. It is unknown whether the impacts of multiple recreational activities occurring in the same area are additive, compounding, or compensatory.

Non-flight energy expenditure, such as increased heart rate, resulting from recreational activities is difficult to measure and, as a result, seldom measured. Although many studies have analyzed the observed reaction distance of bears to varying human activities, the unseen mechanisms of the “fight or flight” response may still lead to an increased energy expenditure for the animal even if they do not exhibit an external reaction [141, 142]. Reynolds et al. [143] showed elevated heart rates in grizzly bears in response to aerial surveys. In addition, effects may last for several days after the encounter and have lingering effects on energy expenditure [80].

Abandonment of dens during hibernation is costly, not only in the movement required to find a second den location but also because the subsequent den quality is often poor (less bedding material and insulation) [26]. Although there have been many documented cases of bears leaving their dens in response to human disturbance, there are potentially other unseen responses, such as increased heart rate, temperature, and activity that are also energetically expensive to a fasting animal and may result in increased weight loss [132, 143, 144, 145, 146]. The susceptibility of denning bears to disturbance must be considered as a function of preferred location and time of year, since most dens were abandoned within two weeks of den entry [26, 146, 147].

### Conclusions

Empirical studies support that one of the primary mechanisms by which bears are affected by recreation is via displacement. Our results, incorporating results of empirical studies and expert knowledge, suggest that displacement may affect individual health and ultimately bear reproduction and survival, primarily as a result of decreased nutritional intake and increased energetic costs. Displacement from concentrated resources often occurs during hyperphagia, when bears dramatically increase their food intake in preparation for hibernation [148, 149]. While empirical studies support that nutritional intake can decline in bears exposed to recreational activities such as bear-viewing and angling, there are no data regarding the impacts of recreations on bear energetic costs. Thus, this may be a particularly important area of future research.

Given that DLPs were frequently mentioned in the literature and by the Delphi Survey Experts, we obtained data from the state of Alaska to include in the BNM. However, the data suggest that the likelihood of a DLP as a result of a recreational activity was so low that we removed DLPs from the model. This is not to say that reducing DLPs are unimportant, but rather that there is not a clear association with recreational activities. Direct mortality as a result of hunting and unreported human-caused mortality was a driving factor in the BNM in the decline in adult survival.

Managers are often required to balance protecting wildlife against providing access for multiple recreational activities. Our results suggest that decisions managers make about regulating recreational activities in time and space have important consequences for bear populations. The BNM provided here is a first step towards providing management with a tool to balance the demands of multiple human recreational activities while supporting healthy bear populations. Furthermore, to reduce the impacts of recreational activities on bears, managers could use the BNM tool to prepare for potential future increases in recreational activities and the expansion of recreational activities to new areas.

## Supporting Information

S1 File PRISMA diagram of the literature search for studies examining the effects of recreation on bears. (DOCX)

S2 File Forms provided to 12 brown bear experts and results from a Delphi survey examining the frequency of recreations in brown bear habitats, potential effects, management suggestions, and research needs. (DOCX)

S3 File Definitions, probability tables, model scenario inputs, and sensitivity analysis results for the Bayesian network model examining the impacts of human recreational activities on Alaskan brown bears. (DOCX)

S4 File User’s manual for the Bayesian network model examining the impacts of human recreational activities on Alaskan brown bears. (DOCX)

S5 File Bayesian network model for assessing the impacts of human recreational activities on Alaskan brown bears. This file can be used in the free version of Netica (Norsys software corp; http://www.norsys.com/download.html). The manual for the model is available in S4 File.
(NETA)
