# Supplementary material for: Impacts of Human Recreation on Brown Bears (Ursus arctos): A Review and New Management Tool
Source: PLoS One. 2016 Jan 5;11(1):e0141983. doi: 10.1371/journal.pone.0141983 (PMC4701408; doi:10.1371/journal.pone.0141983)
Supplement: S1 File — (DOCX) [file pone.0141983.s001.docx]

**S1 File.** PRISMA diagram of the literature search for studies examining the effects of recreation on bears.

PRISMA Flow Diagram:

‘Records screened’, ‘Records excluded’, and ‘Studies included in quantitative synthesis (meta-analysis)’ are not applicable as we were only conducting a literature review not a meta-analysis.

Full-text articles were excluded for the following reasons:

1. Not related to human activities that were not recreational:
   1. Seismic surveys
   2. Hydroelectric
   3. Roads
   4. Mine development
   5. Military training
   6. Open garbage dumps
   7. Developments
   8. Logging
2. Related to conflicts not directly linked to human recreational activities
3. Discussed the location of trails and/or campsites but did not measure the impacts of such activities on brown bears.
4. Analyzed habituation, which we did not include in our discussion because the mechanism by which habituation occurs, the impact to individual bears, and the impact on the population is not known.
5. The impact to denning activity was a direct result of research activity.


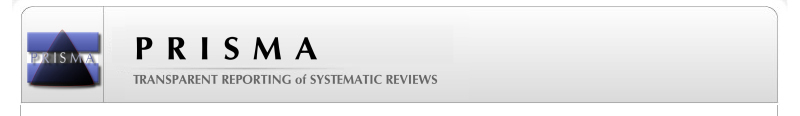
**PRISMA Flow Diagram**

Studies included in quantitative synthesis (meta-analysis)
(n = N/A)

Studies included in qualitative synthesis
(n = 39)

Full-text articles excluded, with reasons
(n = 39)

Full-text articles assessed for eligibility
(n = 85)

Records excluded
(n = N/A )

Records screened
(n = N/A)

Records after duplicates removed
(n = 85)

Additional records identified through other sources
(n = 16)

## Identification

## Eligibility

## Included

## Screening

Records identified through database searching
(n = 74 )
