# Supplementary material for: Impacts of Human Recreation on Brown Bears (Ursus arctos): A Review and New Management Tool
Source: PLoS One. 2016 Jan 5;11(1):e0141983. doi: 10.1371/journal.pone.0141983 (PMC4701408; doi:10.1371/journal.pone.0141983)
Supplement: S2 File — (DOCX) [file pone.0141983.s002.docx]

**S2 File.** Forms provided to 12 brown bear experts and results from a Delphi survey examining the frequency of recreations in brown bear habitats, potential effects, management suggestions, and research needs.

**Round 1 of Delphi Survey**

1. In your opinion what types of human recreation have the greatest levels of disturbance on brown and black bears? Please list and describe each recreation.
2. Can human recreation activities in bear habitats be beneficial to bears? If so, list which activities you believe are beneficial to bears and how they are beneficial.
3. If human recreation is left unmitigated, do you think there are population level impacts on bears? If so, please explain by which mechanisms these population-level effects occur.
4. What management actions are most effective at mitigating potential impacts of human recreation on bears? Please list and describe each management action.
5. What do you see as the most critical knowledge gaps with regards to human recreation and bears?

**Round 2 of Delphi Survey**

1. The table below contains recreations listed by participants to the first round question “In your opinion what types of human recreation have the greatest levels of disturbance on brown and black bears?” Based on your own knowledge and the reasoning given by your fellow participants (given below the table) please rank the human recreations by level of impact for coastal bears with access to salmon and interior bears without, with 1 being the greatest and using each number only once.

| **Human recreation** | **Rank for coastal bears** | **Rank for interior bears** |
| --- | --- | --- |
| Angling |  |  |
| Regulated bear-viewing |  |  |
| Unregulated bear-viewing |  |  |
| Bear hunting |  |  |
| Other hunting |  |  |
| Hiking |  |  |
| Off-trail hiking |  |  |
| Camping |  |  |
| Photography |  |  |
| Tourist developments |  |  |
| Roads |  |  |
| Snow machining |  |  |
| Fixed-winged aircraft |  |  |
| Helicopters |  |  |
| ATV Use |  |  |
| Mushing |  |  |

1. The table below contains the responses from all participants to the first round question “If human recreation is left unmitigated, do you think there are population level impacts on bears?” Based on your own knowledge and the reasoning given by your fellow participants please rank the population level impacts from 1-6, with 1 being the greatest impact and using each number only once.

| **Impact** | **Participants’ reasons** | **Rank** |
| --- | --- | --- |
| Habituation | Bear stop perceiving humans as a threat  Increased risk of mortality |  |
| Reduced survival | Mortality from DLP or food-conditioning  Reduced survival of certain age classes (i.e. subadults)  Increased mortality from motorized vehicles  Improper management of bear hunting |  |
| Decreased nutritional intake | Displacement may lead to acceptance of less nutritious source  Impact on individual health, survival, and reproduction  Improper management of game species |  |
| Habitat decline | Displacement reduces carrying capacity of the environment |  |
| Reduced reproduction | Displacement from mates |  |
| Behavioral changes | Spatial and temporal displacement from avoidance of humans |  |

1. The table below contains the responses from all participants to the first round question “What management actions are most effective at mitigating potential impacts of human recreation on bears?” Based on your own knowledge and the reasoning given by your fellow participants please rank what you feel are the top management actions from 1-5, with 1 being the most effective and using each number only once.

| Management action | Participants’ reasons | Rank |
| --- | --- | --- |
| Educate the public | Prevents conflict and disturbance  Reduces food-conditioning when food and garbage are stored properly  Proper use of bear-spray  Use of electric fencing for camps  Safe recreation in bear country |  |
| Regulation of angling | Closures of prime areas  Restrictions on where to clean fish  Proper fish storage to minimize bears obtaining fish from anglers  Require knowledgeable guides |  |
| Regulate bear-viewing | Minimize direct interactions (i.e. elevated platforms)  Minimize habituation  Require knowledgeable guides |  |
| Seasonal closures of high density bear areas | Reduce displacement  Reduce encounters and confrontations  Reduce stress caused by human recreation |  |
| Hunting regulations | Regulate for access  Educate hunters to species identification to reduce taking of grizzly bears identified as black bears  Educate to reduce conflicts with bears  Sustainable harvest |  |
| Create and reinforce existing protected areas | Areas free of human activity to protect prime habitat |  |
| Control food and garbage storage | Prevents food-conditioned bears, minimizing conflicts and reducing bear removals |  |
| Campground and trail placement | Reduce placement in areas if important food resources |  |
| Temporally control access | Allow predictable times of day free from humans |  |
| Road density | Manage to limit motorized routes in prime habitat |  |

1. The list below contains the responses from all participants to the first round question “What do you see as the most critical knowledge gaps with regards to human recreation and bears?” Please weight the importance of each item below with a value from 1-10, with 1 being the most important and 10 the least.
   1. Effects of human recreation on bear reproduction
   2. Effects of disturbance on fitness
   3. Effects of human recreation on nutritional intake
   4. Effects of snow machining on denning bears
   5. Displacement of bears by ATV activity
   6. Displacement from salmon streams by fishermen
   7. Identify prime habitats for human exclusion
   8. Identify distances at which humans don’t disturb bears
   9. Evaluate population impacts from human recreation
   10. Identify thresholds for number of humans recreating
   11. Identify what is happening in the back country or at coastal sites

as most studies are done in prime bear-viewing areas

- 1. Identify if and what the relationship is between habituation, displacement,

and stress caused by human recreation

- 1. Conduct site-specific research and guidance - “one size fits all” does

not work aside from basic advice

- 1. Develop improved methods for hunters to carry and use bear spray

while carrying firearms

- 1. Identify landscape wide distribution of age classes due to human avoidance
  2. Identify and regulating problem behavior by humans specific to

sites (i.e. camping in high density bear areas without perimeter fencing)

- 1. Identify habitat types associated with increased bear-human conflict
  2. Density estimation
  3. People’s attitudes towards bears and tolerance levels
  4. Identify and quantify the number of people participating in recreational

activities in bear habitat.

**Round 3 of Delphi Survey**

PLEASE NOTE: This survey round includes a request for additional information on recreational activities that were most common and/or perceived to have the greatest impact. Some human recreations and factors affecting access to recreational areas discussed in previous surveys are not discussed here but will be discussed in the manuscript per your prior input.

Two tables are provided below: one requesting input on coastal populations and one on interior populations. Please respond to one or both dependent on your experience with populations in these categories. Note that your responses should be in regards to populations in which you have specific experience. Geographic and population-level variation will be accounted for via our selection of participants in the panel.

DEFINITIONS: Note that “regulated bear-viewing” refers to limitations on viewers in time and space (e.g., Anan Creek, McNeil River) as opposed to unregulated bear-viewing where viewers are not required to use specific viewing areas and access to the area is not limited to set hours.

STEP 1: In the following tables, use the categories below to indicate in the first column how common each type of recreation is across the populations you are familiar with.

0: Does not occur

1: Rare

2: Common

3: Very common

STEP 2: In the second column, use the categories below to estimate the proportion of the population that is exposed to the recreational activity listed:

0: Does not occur

1: 0-5%

2: 5-35%

3: 35-65%

4: 65-95%

5: 95-100%

STEP 3: Under the third column “impacts on segment of population exposed” check each category of impact you believe has the ability to effect the population if that human recreation is left unmitigated. NOTE: Reduced survival should include consideration of direct mortalities due to defense of life and property.

1. **Coastal bears**

| Human recreation | Frequency of recreation | Percentage population affected | Potential impacts on segment of population exposed | | | |
| --- | --- | --- | --- | --- | --- | --- |
|  |  |  | Reduced survival | Decreased nutritional intake | Displacement | Reduced reproduction |
| Angling |  |  |  |  |  |  |
| Regulated bear-viewing |  |  |  |  |  |  |
| Unregulated bear-viewing |  |  |  |  |  |  |
| Bear hunting |  |  |  |  |  |  |
| Other hunting |  |  |  |  |  |  |
| Hiking |  |  |  |  |  |  |
| Off-trail hiking |  |  |  |  |  |  |
| Camping |  |  |  |  |  |  |
| Photography |  |  |  |  |  |  |
| Helicopters |  |  |  |  |  |  |
| Snow machining |  |  |  |  |  |  |
| Fixed-winged aircraft |  |  |  |  |  |  |
| ATV Use |  |  |  |  |  |  |

**Comments:**

1. **Interior bears**

| Human recreation | Frequency of recreation | Percentage population affected | Impacts on segment of population exposed | | | |
| --- | --- | --- | --- | --- | --- | --- |
|  |  |  | Reduced survival | Decreased nutritional intake | Displacement | Reduced reproduction |
| Angling |  |  |  |  |  |  |
| Regulated bear-viewing |  |  |  |  |  |  |
| Unregulated bear-viewing |  |  |  |  |  |  |
| Bear hunting |  |  |  |  |  |  |
| Other hunting |  |  |  |  |  |  |
| Hiking |  |  |  |  |  |  |
| Off-trail hiking |  |  |  |  |  |  |
| Camping |  |  |  |  |  |  |
| Photography |  |  |  |  |  |  |
| Helicopters |  |  |  |  |  |  |
| Snow machining |  |  |  |  |  |  |
| Fixed-winged aircraft |  |  |  |  |  |  |
| ATV Use |  |  |  |  |  |  |

**Comments:**

Table A. The mean weight (with 1 being the most important) and standard deviation (SD) of the effectiveness of management actions to minimize the impacts of recreational activities on bears identified by 12 experts during a modified Delphi survey. Management actions were ranked by the most important to mitigate for the potential impacts of human recreational activities on bears, with 1 being the most effective.

| Management action | Participants’ reasons | Mean | SD |
| --- | --- | --- | --- |
| Educate the public | Prevents conflict and disturbance  Reduces food-conditioning when food and garbage are stored properly  Proper use of bear-spray  Promotes use of electric fencing for camps  Promotes safe recreation in bear country | 2.8 | 2.3 |
| Control food and garbage storage | Prevents food-conditioned bears, minimizing conflicts and reducing bear removals | 3.2 | 1.7 |
| Road density | Manage to limit motorized routes in prime habitat | 3.6 | 2.7 |
| Hunting regulations | Regulate for access  Educate hunters to species identification to reduce taking of grizzly bears identified as black bears  Educate to reduce conflicts with bears  Promotes sustainable harvest | 4.3 | 3.1 |
| Create and reinforce existing protected areas | Creates areas free of human activity to protect prime habitat | 4.7 | 2.4 |
| Seasonal closures of high density bear areas | Reduces displacement  Reduces encounters and confrontations  Reduces stress caused by human recreation | 5.3 | 1.9 |
| Campground and trail placement | Reduces placement in areas if important food resources | 6.0 | 1.9 |
| Regulate bear-viewing | Minimizes direct interactions (i.e. elevated platforms)  Minimizes habituation  Requires knowledgeable guides | 6.1 | 2.8 |
| Regulation of angling | Closures of prime areas  Restrictions on where to clean fish  Promotes proper fish storage to minimize bears obtaining fish from anglers  Requires knowledgeable guides | 6.2 | 2.8 |
| Temporally control access | Allows predictable times of day free from humans | 7.5 | 2.1 |

Table B. The mean weight (with 1 being the most important) and standard deviation (SD) prioritizing research to fill the current knowledge gaps regarding the impacts of recreational activities on bears identified by 12 experts during a modified Delphi survey.

| Research objective | Mean | SD |
| --- | --- | --- |
| Identify if and what the relationship is between habituation, displacement, and stress caused by human recreation | 3.2 | 2.4 |
| Identify people's attitudes towards bears and tolerance levels | 3.3 | 2.5 |
| Assess displacement from salmon streams by fishermen | 3.4 | 1.6 |
| Evaluate population impacts from human recreation | 3.6 | 3.1 |
| Identify prime habitats for human exclusion | 3.7 | 1.7 |
| Identify and quantify the number of people participating in recreational activities in bear habitat | 3.7 | 1.6 |
| Examine the effects of human recreation on nutritional intake | 3.9 | 1.6 |
| Conduct site-specific research and guidance - "one size fits all" does not work aside from basic advice | 4.0 | 2.7 |
| Identify habitat types associated with increased bear-human conflict | 4.0 | 2.1 |
| Evaluate the effects of disturbance on fitness | 4.1 | 2.8 |
| Identify distances at which humans don't disturb bears | 4.3 | 2.6 |
| Develop improved methods for hunters to carry and use bear spray while carrying firearms | 4.4 | 2.7 |
| Assess the effects of human recreation on bear reproduction | 4.5 | 2.2 |
| Identify and regulate problem behavior by humans specific to sites (i.e. camping in high density bear areas) | 4.7 | 2.6 |
| Identify thresholds for number of humans recreating | 4.8 | 2.7 |
| Evaluate displacement of bears by ATV activity | 4.8 | 2.3 |
| Identify landscape wide distributions of age classes due to human avoidance | 5.2 | 2.4 |
| Identify what is happening in the back country or at coastal sites (non-prime bear-viewing areas) | 5.3 | 2.1 |
| Evaluate the effects of snow machining on denning bears | 5.3 | 2.6 |
| Conduct a density estimation | 5.6 | 2.8 |
