# Supplementary material for: Impacts of Human Recreation on Brown Bears (Ursus arctos): A Review and New Management Tool
Source: PLoS One. 2016 Jan 5;11(1):e0141983. doi: 10.1371/journal.pone.0141983 (PMC4701408; doi:10.1371/journal.pone.0141983)
Supplement: S3 File — (DOCX) [file pone.0141983.s003.docx]

**S3 File.** Definitions, probability tables, model scenario inputs, and sensitivity analysis results for the Bayesian network model examining the impacts of human recreational activities on Alaskan brown bears.

Table A. Bayesian network impacts of recreational activities on Alaskan brown bears model input, intermediate, and output nodes titles, descriptions, and states. The measurement for all states is the annual user days or user nights per square mile.

| Node name | Node title | Node description | States |
| --- | --- | --- | --- |
| *Input node: unregulated activities & dispersed resources* | | | |
| unhike | Unregulated hiking | Hiking that is not restricted to trails or regulations on limiting use to trails are unenforced. | None (0)  Low (>0 to <1)  Medium (>=1 to <3)  High (≥3) |
| unATV | Unregulated ATV use | ATV use that is not restricted to trails or regulations limiting use to trails are unenforced. | None (0)  Low (>0 to <1)  Medium (≥1 to <3)  High (≥3) |
| undispc | Unregulated, dispersed camping | Camping where resources are dispersed, that occurs in a dispersed manner (not in regulated campsites), and regulations on proper food and garbage do not exist or are unenforced. | None (0)  Uncommon (>0 to <0.1)  Common (≥0.1) |
| uncampc | Unregulated, campground camping | Camping in campgrounds where resources are dispersed and regulations on proper storage of food and garbage do not exist or are unenforced. | None (0)  Low (>0 to <1)  Medium (≥1 to <10)  High (≥10) |
| H2O | Non-motorized water recreation | Non-motorized water recreation (i.e. rafting, kayaking). | None (0)  Low (>0 to <1)  Medium (≥1 to <2)  High (≥2) |
| *Input node: regulated activities & dispersed resources* | | | |
| Dispc | Regulated, dispersed camping | Camping where resources are dispersed, camping occurs in a dispersed manner (not in regulated campsites), and regulations are in place and enforced for proper storage of food and garbage. | None (0)  Uncommon (>0 to <0.1)  Common (≥0.1) |
| Campc | Regulated, campground camping | Camping in campgrounds where resources are dispersed and regulations are in place and enforced for proper storage of food and garbage. | None (0)  Low (>0 to <1)  Medium (≥1 to <10)  High (≥10) |
| trail | Trail use | Use of trails where resources are dispersed, includes horseback riding, hiking, biking, and trail-running with enforced regulations to trail use. | None (0)  Low (>0 to <10)  Medium (≥10 to <20)  High (≥20) |
| ATV | Trail ATV use | ATV use is restricted to trails and the regulation is enforced. | None (0)  Low (>0 to <1)  Medium (≥1 to <3)  High (≥3) |
| *Input node: regulated activities & concentrated resources* | | | |
| dispc2 | Regulated, dispersed camping | Camping where resources are concentrated, camping occurs in a dispersed manner (not in regulated campsites), and regulations are in place and enforced for proper storage of food and garbage. | None (0)  Uncommon (>0 to <0.1)  Common (≥0.1) |
| campc2 | Regulated, campground camping | Camping in campgrounds where resources are concentrated and regulations are in place and enforced for proper storage of food and garbage. | None (0)  Low (>0 to <1)  Medium (≥1 to <10)  High (≥10) |
| trail2 | Trail use | Use of trails where resources are concentrated, includes horseback riding, hiking, biking, and trail-running with enforced regulations to trail use. | None (0)  Low (>0 to <10)  Medium (≥10 to <20)  High (≥20) |
| bv | Regulated bear-viewing | Bear-viewing is regulated spatially and temporally and regulations are enforced. | None (0)  Low (>0 to <3)  Medium (≥3 to <10)  High (≥10) |
| angst | Regulated angling – spatial/temporal | Hours and/or locations are limited during which anglers can fish. | None (0)  Low (>0 to <1)  Medium (≥1 to <20)  High (≥20) |
| angfh | Regulated angling – fish handling | Regulations are enforced for cleaning and storage of fish. | None (0)  Low (>0 to <1)  Medium (≥1 to <20)  High (≥20) |
| *Input node: unregulated activities & concentrated resources* | | | |
| undispc2 | Unregulated, dispersed camping | Camping where resources are concentrated, camping occurs in a dispersed manner (not in regulated campsites), and regulations on proper storage of food and garbage do not exist or are unenforced. | None (0)  Uncommon (>0 to <0.1)  Common (≥0.1) |
| uncampc2 | Unregulated, campground camping | Camping in campgrounds where resources are concentrated and regulations on proper storage of food and garbage do not exist or are unenforced. | None (0)  Low (>0 to <1)  Medium (≥1 to <10)  High (≥10) |
| unbv | Unregulated bear-viewing & photography | Bear-viewers and photographers that are not restricted temporally or spatially or where regulations are unenforced. | None (0)  Low (>0 to <3)  Medium (≥3 to <10)  High (≥10) |
| unangst | Unregulated angling – spatial/temporal | No spatial or temporal limitations on river access for anglers. | None (0)  Low (>0 to <1)  Medium (≥1 to <20)  High (≥20) |
| unangfh | Unregulated angling – fish handling | No regulations or regulations are unenforced for fish cleaning and storage. | None (0)  Low (>0 to <1)  Medium (≥1 to <20)  High (≥20) |
| *Input nodes: winter recreation* | | | |
| nmotw | Non-motorized winter recreation | Recreational activities that occur during bear denning that are non-motorized to include cross-country skiing, downhill skiing (including heli-skiing), snow shoeing, and dog sledding. Helicopter access for heli-skiing was not considered as it is an access issue. | None (0)  Uncommon (>0 to <1)  Common (≥1) |
| motw | Motorized winter recreation | Recreational activities that occur during bear denning that are motorized to include snow machining. | None (0)  Uncommon (>0 to <1)  Common (≥1) |
| *Other input nodes* | | | |
| ohunt | Other hunting/non-winter trapping | Hunting and non-winter trapping for any game animals other than brown bears. All activities associated with other hunting (i.e. ATV use) should be included here rather than in other recreational activity nodes. | None (0)  Low (>0 to <1)  Medium (≥1 to <3)  High (≥3) |
| bbh | Brown bear harvest | Any legal take of brown bears to include bear hunting, incidental take, defense of life and property kills, and removal due to management action. Measured by the management goal. | None  Reduction  Sustained yield  Recovery |
| unreport | Unreported human-caused mortality | Brown bear kills that go unreported (i.e. unreported DLPs and poaching). This node is left to the discretion of the manager as data is not usually available as to the number of unreported brown bear kills. Managers should consider the percentage of the population they estimate are impacted, the density of the population, and the reproductive rate of the population. For example, 4% unreported mortality in an interior population such as Denali NP has a higher impact than to a coastal population such as Katmai NP. | Not influential  Influential |
| *Intermediate child nodes* | | | |
| lowhab | Displacement from low quality habitat | Temporal or spatial avoidance of humans in habitat that does not contain high quality and/or concentrated food resources (i.e. moose or cow parsnip). Berries may be considered as either a dispersed or concentrated food resource depending on the concentration on the landscape, type of berry, and if it’s a good or bad berry year. | None  Temporary  Long-term |
| highhab | Displacement from high quality habitat | Temporal or spatial avoidance of humans in habitat that contains high quality and/or concentrated food resources (i.e. salmon streams) when no alternatives are available within the average daily movement of a brown bear. Berries may be considered as either a dispersed or concentrated food resource depending on the concentration on the landscape, type of berry, and if it’s a good or bad berry year. | None  Temporary  Long-term |
| nutr | Nutritional intake | Percent increase or decrease in nutritional intake, either in the amount of food consumed (i.e. decreased time spent fishing when humans present) or in the quality of food consumed. | Percent decreased  No change from baseline  Percent increased |
| ecost | Energetic costs | Percent increase in energy expenditure as a result of recreational activities (i.e. increased heart rate, movement away). Does not include changes due to decreased nutritional intake (i.e. decreased time spent feeding). | No change from baseline  Percent increased |
| *Output nodes* | | | |
| repro | Reproduction | Percent increase or decrease in reproductive success (i.e. having cubs) as a result of changed nutritional intake or energetic costs that may lead to a change in maternal body condition. | Percent decreased  No change from baseline  Percent increased |
| cub | Cub survival | Percent increase or decrease in cub survival as a result of den abandonment, change in direct nutritional intake or energetic costs, or change in maternal body condition. | Percent decreased  No change from baseline  Percent increased |
| adult | Adult survival | Percent increase or decrease in adult survival as a result of direct mortality (i.e. brown bear harvest or unreported human-caused mortality), decreased body condition (i.e. decreased nutritional intake or increased energetic costs), or unreported human-caused mortality. | Percent decreased  No change from baseline  Percent increased |

Table B. Examples of user levels by recreational activity type. The same examples are used in order to give a common reference, not because the activity for that area is unregulated versus regulated activities or occurs in dispersed or concentrated resources. All recreational activities were measured in annual user days or user nights and expressed as number per square mile because the size of jurisdictions for different managers may vary. NF = national forest; NP = national park; NPP = national park & preserve; NWR = national wildlife refuge

| Recreational activity | Node state | Example |
| --- | --- | --- |
| Off-trail Hiking | Low (>0 to <1)  Medium (≥1 to <3)  High (≥3) | Tongass NF; Gates of the Arctic NPP  Chugach NF |
| ATV use | Low (>0 to <1)  Medium (≥1 to <3)  High (≥3) | Tongass NF; Chugach NF |
| Dispersed camping | Uncommon (>0 to <0.1)  Common (≥0.1) | Katmai NPP  Denali NP |
| Campground camping | Low (>0 to <1)  Medium (≥1 to <10)  High (≥10) | Katmai NPP  Tongass NF  Chugach NF; Denali NP |
| Non-motorized water recreation | Low (>0 to <1)  Medium (≥1 to <2)  High (≥2) | Kodiak NWR  Tongass NF  Chugach NF |
| Trail use | Low (>0 to <10)  Medium (≥10 to <20)  High (≥20) | Tongass NF Sitka & Hoonah districts  Tongass NF Juneau & Admiralty districts; Chugach NF |
| Bear-viewing | Low (>0 to <3)  Medium (≥3 to <10)  High (≥10) | McNeil River Falls  Brooks Falls in Katmai National Park |
| Angling | Low (>0 to <1)  Medium (≥1 to <20)  High (≥20) | McNeil River State Game Sanctuary  Chugach NF; Tongass NF  Kenai-Russian River Area |
| Non-motorized winter recreation | Uncommon (>0 to <1)  Common (≥1) | Tongass NF  Chugach NF |
| Motorized winter recreation | Uncommon (>0 to <1)  Common (≥1) | Kenai Fjords NP  Chugach NF |
| Other hunting/non-winter trapping | Low (>0 to <1)  Medium (≥1 to <3)  High (≥3) | Katmai NPP; Tongass NF Sitka & Hoonah districts  Chugach NF; Tongass NF Juneau & Admiralty districts  Tongass NF Yakutat, Petersburg & Wrangell districts |

Table C. The index of impact for each recreational activity, developed with group consensus. A value of 1-5 (1 = lowest impact and 5 = highest impact) was assigned to each recreational activity and state combination based on the relative impact compared to other recreational activities. If a recreational activity did not occur then it was assigned an index value of zero.

| Summary node | Recreation | None | Low | Medium | High |
| --- | --- | --- | --- | --- | --- |
| Unregulated activities & dispersed resources | Unregulated hiking | 0 | 1 | 1 | 2 |
|  | Unregulated ATV use | 0 | 1 | 2 |  |
|  | Unregulated dispersed camping | 0 | 2 | 2 |  |
|  | Unregulated campground camping | 0 | 2 | 2 | 3 |
|  | Non-motorized water recreation | 0 | 1 | 1 | 1 |
| Regulated activities & dispersed resources | Regulated dispersed camping | 0 | 1 | 1 |  |
|  | Regulated campground camping | 0 | 1 | 1 | 1 |
|  | Trail use | 0 | 1 | 2 | 2 |
|  | Trail ATV use | 0 | 1 | 2 |  |
| Regulated activities & concentrated resources | Regulated dispersed camping | 0 | 1 | 2 |  |
|  | Regulated campground camping | 0 | 2 | 2 | 3 |
|  | Trail use | 0 | 2 | 2 | 3 |
|  | Regulated bear-viewing | 0 | 1 | 2 | 2 |
|  | Regulated angling - spatial/temporal | 0 | 1 | 2 | 2 |
|  | Regulated angling - fish handling | 0 | 1 | 2 | 3 |
| Unregulated activities & concentrated resources | Unregulated dispersed camping | 0 | 2 | 3 |  |
|  | Unregulated campground camping | 0 | 3 | 3 | 4 |
|  | Unregulated bear-viewing & photography | 0 | 2 | 3 | 3 |
|  | Unregulated angling - spatial/temporal | 0 | 2 | 3 | 3 |
|  | Unregulated angling - fish handling | 0 | 2 | 3 | 4 |
| Winter recreation | Non-motorized winter | 0 | 1 | 1 |  |
|  | Motorized winter | 0 | 1 | 1 |  |
|  | Other hunting/non-winter trapping | 0 | 2 | 2 | 3 |
|  | Bear harvest | 0 | 1 | 1 | 5 |

Table D. The states for the summary nodes (i.e. unregulated activities & dispersed resources) are no recreational activity, low impact, medium impact, and high impact. To assign impact levels at the summary nodes, first the possible points for that node were calculated (i.e. the number of recreational activities * 5).  Next the maximum number of points were divided into thirds with the lowest third assigned to low impact, the medium third to medium impact, and the highest third to high impact.

| Summary node | Max | Low Impact | Medium Impact | High Impact |
| --- | --- | --- | --- | --- |
| Unregulated activities & dispersed resources | 25 | 1-8 | 9-16 | 17-25 |
| Regulated activities & dispersed resources | 20 | 1-6 | 7-13 | 14-20 |
| Regulated activities & concentrated resources | 30 | 1-10 | 11-20 | 21-30 |
| Unregulated activities & concentrated resources | 25 | 1-8 | 9-16 | 17-25 |
| Winter recreation | 10 | 1-3 | 4-6 | 7-10 |
| Other hunting/non-winter trapping | 5 | 1 | 2-3 | 4-5 |
| Bear harvest | 5 | 1 | 2-3 | 4-5 |

Table E. Conditional probability tables for each intermediate and output node for the Bayesian network impacts of recreational activities on Alaskan brown bears model. Probability tables for the input nodes are not shown as they are uniform probability distributions unless level of use is specified by the user.

Table E1. Probability table for the node ‘Displacement from low quality habitat’.

| Regulated activity in dispersed resources^a^ | Other hunting/non-winter trapping | Unregulated activity in dispersed activity^a^ | None | Temporary | Long-term |
| --- | --- | --- | --- | --- | --- |
| None | None | None | 100 | 0 | 0 |
| None | None | Low | 94 | 6 | 1 |
| None | None | Medium | 86 | 12 | 2 |
| None | None | High | 78 | 17 | 5 |
| None | Low | None | 93 | 7 | 0 |
| None | Low | Low | 89 | 10 | 1 |
| None | Low | Medium | 81 | 15 | 4 |
| None | Low | High | 75 | 18 | 7 |
| None | Medium | None | 89 | 11 | 1 |
| None | Medium | Low | 86 | 12 | 2 |
| None | Medium | Medium | 78 | 18 | 4 |
| None | Medium | High | 72 | 22 | 7 |
| None | High | None | 83 | 15 | 2 |
| None | High | Low | 78 | 19 | 3 |
| None | High | Medium | 72 | 23 | 5 |
| None | High | High | 67 | 25 | 8 |
| Low | None | None | 95 | 4 | 0 |
| Low | None | Low | 87 | 13 | 1 |
| Low | None | Medium | 80 | 18 | 2 |
| Low | None | High | 74 | 20 | 6 |
| Low | Low | None | 92 | 7 | 0 |
| Low | Low | Low | 84 | 15 | 2 |
| Low | Low | Medium | 76 | 21 | 4 |
| Low | Low | High | 71 | 22 | 7 |
| Low | Medium | None | 85 | 14 | 1 |
| Low | Medium | Low | 79 | 20 | 2 |
| Low | Medium | Medium | 74 | 22 | 4 |
| Low | Medium | High | 69 | 24 | 7 |
| Low | High | None | 80 | 17 | 3 |
| Low | High | Low | 75 | 22 | 3 |
| Low | High | Medium | 69 | 24 | 7 |
| Low | High | High | 63 | 28 | 9 |
| Medium | None | None | 87 | 10 | 3 |
| Medium | None | Low | 81 | 16 | 3 |
| Medium | None | Medium | 75 | 19 | 5 |
| Medium | None | High | 66 | 23 | 10 |
| Medium | Low | None | 80 | 17 | 3 |
| Medium | Low | Low | 76 | 20 | 4 |
| Medium | Low | Medium | 70 | 24 | 6 |
| Medium | Low | High | 62 | 27 | 11 |
| Medium | Medium | None | 76 | 20 | 5 |
| Medium | Medium | Low | 71 | 23 | 6 |
| Medium | Medium | Medium | 64 | 29 | 7 |
| Medium | Medium | High | 57 | 32 | 11 |
| Medium | High | None | 72 | 23 | 5 |
| Medium | High | Low | 67 | 28 | 5 |
| Medium | High | Medium | 60 | 33 | 7 |
| Medium | High | High | 53 | 35 | 12 |
| High | None | None | 79 | 18 | 3 |
| High | None | Low | 74 | 23 | 4 |
| High | None | Medium | 65 | 27 | 7 |
| High | None | High | 61 | 28 | 11 |
| High | Low | None | 74 | 20 | 5 |
| High | Low | Low | 68 | 26 | 6 |
| High | Low | Medium | 60 | 30 | 10 |
| High | Low | High | 53 | 34 | 13 |
| High | Medium | None | 67 | 27 | 7 |
| High | Medium | Low | 62 | 31 | 7 |
| High | Medium | Medium | 52 | 37 | 11 |
| High | Medium | High | 46 | 38 | 15 |
| High | High | None | 58 | 34 | 8 |
| High | High | Low | 49 | 39 | 11 |
| High | High | Medium | 42 | 45 | 13 |
| High | High | High | 33 | 49 | 17 |

a: ‘None’ = No_recreational_activity; ‘Low’ = Low_impact; ‘Medium’ = Medium_impact; ‘High’ = High_impact

Table E2. Probability table for the node ‘Displacement from high quality habitat’.

| Unregulated activities in concentrated resources^a^ | Regulated activities in concentrated resources^a^ | None | Temporary | Long-term |
| --- | --- | --- | --- | --- |
| None | None | 100 | 0 | 0 |
| None | Low | 80 | 17.5 | 2.5 |
| None | Medium | 65 | 28.5 | 6.5 |
| None | High | 59 | 30.25 | 10.75 |
| Low | None | 74 | 22.5 | 3.5 |
| Low | Low | 73 | 22 | 5 |
| Low | Medium | 65.25 | 27.5 | 7.25 |
| Low | High | 65.5 | 27.25 | 7.25 |
| Medium | None | 69.75 | 22 | 8.25 |
| Medium | Low | 60.5 | 28.5 | 11 |
| Medium | Medium | 52 | 35.25 | 12.75 |
| Medium | High | 45.25 | 38.75 | 16 |
| High | None | 47 | 39.75 | 13.25 |
| High | Low | 35.25 | 48.75 | 16 |
| High | Medium | 24.75 | 56.25 | 19 |
| High | High | 18.75 | 55.75 | 25.5 |

a: ‘None’ = No_recreational_activity; ‘Low’ = Low_impact; ‘Medium’ = Medium_impact; ‘High’ = High_impact

Table E3. Probability table for the node ‘Nutritional intake’.

| Displacement from low quality habitat | Displacement from high quality habitat | Other hunting/non-winter trapping | Decreased | | | | | | |  | Increased | |
| --- | --- | --- | --- | --- | --- | --- | --- | --- | --- | --- | --- | --- |
|  |  |  | 60-70 | 50-60 | 40-50 | 30-40 | 20-30 | 10-20 | 1-10 | None | 1-10 | 10-20 |
| None | None | None | 0 | 0 | 0 | 0 | 0 | 0 | 0 | 100 | 0 | 0 |
| None | None | Low | 0 | 0 | 0 | 0 | 0 | 0 | 0 | 40 | 60 | 0 |
| None | None | Medium | 0 | 0 | 0 | 0 | 0 | 0 | 20 | 0 | 80 | 0 |
| None | None | High | 0 | 0 | 0 | 0 | 0 | 0 | 20 | 0 | 40 | 40 |
| None | Temporary | None | 0 | 0 | 0 | 0 | 20 | 60 | 20 | 0 | 0 | 0 |
| None | Temporary | Low | 0 | 0 | 0 | 0 | 0 | 80 | 20 | 0 | 0 | 0 |
| None | Temporary | Medium | 0 | 0 | 0 | 0 | 20 | 60 | 0 | 0 | 20 | 0 |
| None | Temporary | High | 0 | 0 | 0 | 0 | 20 | 20 | 40 | 0 | 20 | 0 |
| None | Long-term | None | 0 | 20 | 40 | 0 | 20 | 20 | 0 | 0 | 0 | 0 |
| None | Long-term | Low | 0 | 0 | 40 | 20 | 0 | 40 | 0 | 0 | 0 | 0 |
| None | Long-term | Medium | 0 | 0 | 40 | 20 | 0 | 20 | 20 | 0 | 0 | 0 |
| None | Long-term | High | 0 | 0 | 40 | 20 | 0 | 0 | 20 | 20 | 0 | 0 |
| Temporary | None | None | 0 | 0 | 0 | 0 | 0 | 60 | 40 | 0 | 0 | 0 |
| Temporary | None | Low | 0 | 0 | 0 | 0 | 0 | 40 | 60 | 0 | 0 | 0 |
| Temporary | None | Medium | 0 | 0 | 0 | 0 | 0 | 40 | 40 | 0 | 20 | 0 |
| Temporary | None | High | 0 | 0 | 0 | 0 | 0 | 20 | 40 | 0 | 40 | 0 |
| Temporary | Temporary | None | 0 | 0 | 0 | 40 | 40 | 0 | 20 | 0 | 0 | 0 |
| Temporary | Temporary | Low | 0 | 0 | 0 | 20 | 40 | 20 | 20 | 0 | 0 | 0 |
| Temporary | Temporary | Medium | 0 | 0 | 0 | 20 | 40 | 20 | 20 | 0 | 0 | 0 |
| Temporary | Temporary | High | 0 | 0 | 0 | 20 | 40 | 20 | 0 | 0 | 20 | 0 |
| Temporary | Long-term | None | 0 | 60 | 0 | 0 | 40 | 0 | 0 | 0 | 0 | 0 |
| Temporary | Long-term | Low | 0 | 40 | 20 | 0 | 40 | 0 | 0 | 0 | 0 | 0 |
| Temporary | Long-term | Medium | 0 | 40 | 20 | 0 | 20 | 20 | 0 | 0 | 0 | 0 |
| Temporary | Long-term | High | 0 | 20 | 40 | 0 | 0 | 40 | 0 | 0 | 0 | 0 |
| Long-term | None | None | 0 | 0 | 0 | 0 | 20 | 80 | 0 | 0 | 0 | 0 |
| Long-term | None | Low | 0 | 0 | 0 | 0 | 0 | 80 | 20 | 0 | 0 | 0 |
| Long-term | None | Medium | 0 | 0 | 0 | 0 | 20 | 40 | 40 | 0 | 0 | 0 |
| Long-term | None | High | 0 | 0 | 0 | 0 | 20 | 20 | 20 | 20 | 20 | 0 |
| Long-term | Temporary | None | 0 | 0 | 20 | 40 | 20 | 20 | 0 | 0 | 0 | 0 |
| Long-term | Temporary | Low | 0 | 0 | 0 | 40 | 40 | 20 | 0 | 0 | 0 | 0 |
| Long-term | Temporary | Medium | 0 | 0 | 0 | 40 | 40 | 0 | 20 | 0 | 0 | 0 |
| Long-term | Temporary | High | 0 | 0 | 0 | 40 | 20 | 20 | 20 | 0 | 0 | 0 |
| Long-term | Long-term | None | 40 | 20 | 0 | 20 | 20 | 0 | 0 | 0 | 0 | 0 |
| Long-term | Long-term | Low | 20 | 40 | 0 | 0 | 40 | 0 | 0 | 0 | 0 | 0 |
| Long-term | Long-term | Medium | 40 | 20 | 0 | 0 | 20 | 20 | 0 | 0 | 0 | 0 |
| Long-term | Long-term | High | 20 | 40 | 0 | 0 | 0 | 40 | 0 | 0 | 0 | 0 |

Table F. Inputs nodes for Bayesian network model impacts of recreational activities on Alaskan brown bears to compare the impacts of recreational activities that are 1) unregulated and in dispersed resources; 2) regulated and in dispersed resources; 3) regulated and in concentrated resources; 4) unregulated and in concentrated resources; and 5) all recreational activities occurring simultaneously.

| Node title | 1 | | 2 | | 3 | | 4 | 5 |
| --- | --- | --- | --- | --- | --- | --- | --- | --- |
| ***Input nodes: unregulated activities & dispersed resources*** | | | | | | | | |
| Unregulated hiking | High | | None | | None | | None | High |
| Unregulated ATV use | High | | None | | None | | None | High |
| Unregulated, dispersed camping | Common | | None | | None | | None | Common |
| Unregulated, campground camping | High | | None | | None | | None | High |
| Non-motorized water recreation | High | | None | | None | | None | High |
| ***Input nodes: regulated activities & dispersed resources*** | | | | | | | | |
| Regulated, dispersed camping | None | | High | | None | | None | High |
| Regulated, campground camping | None | | Common | | None | | None | Common |
| Trail use | None | | High | | None | | None | High |
| Trail ATV use | None | | High | | None | | None | High |
| ***Input nodes: regulated activities & concentrated resources*** | | | | | | | | |
| Regulated, dispersed camping | None | | None | | Common | | None | Common |
| Regulated, campground camping | None | | None | | High | | None | High |
| Trail use | None | | None | | High | | None | High |
| Regulated bear-viewing | None | | None | | High | | None | High |
| Regulated angling – spatial/temporal | None | | None | | High | | None | High |
| Regulated angling – fish handling | None | | None | | High | | None | High |
| ***Input nodes: unregulated activities & concentrated resources*** | | | | | | | | |
| Unregulated, dispersed camping | None | | None | | None | | Common | Common |
| Unregulated, campground camping | None | | None | | None | | High | High |
| Unregulated bear-viewing & photography | None | | None | | None | | High | High |
| Unregulated angling – spatial/temporal | None | | None | | None | | High | High |
| Unregulated angling – fish handling | None | | None | | None | | High | High |
| ***Input nodes: winter recreation*** | | | | | | | | |
| Non-motorized winter recreation | None | | None | | None | | None | Common |
| Motorized winter recreation | None | | None | | None | | None | Common |
| ***Other input nodes*** | | | | | | | | |
| Other hunting/non-winter trapping | | None | | None | | None | None | High |
| Brown bear harvest | | None | | None | | None | None | Reduction |
| Unreported human-caused mortality | | Not influential | | Not influential | | Not influential | Not influential | Influential |

Table G. Inputs nodes for Bayesian network model impacts of recreational activities on Alaskan brown bears for the management areas: 1) Brooks Camp in Katmai National Park and Preserve; 2) Hallo Bay in Katmai National Park & Preserve; and 3) the Kenai-Russian River Management Area.

| Node title | Scenario state levels | | | | | |
| --- | --- | --- | --- | --- | --- | --- |
|  | Brooks Camp | | Hallo Bay | | Russian River | |
| ***Input nodes: unregulated activities & dispersed resources*** | | | | | | |
| Unregulated hiking | | Low | | Medium | | High |
| Unregulated ATV use | | None | | None | | None |
| Unregulated, dispersed camping | | None | | None | | None |
| Unregulated, campground camping | | None | | None | | None |
| Non-motorized water recreation | | Low | | None | | Medium |
| ***Input nodes: regulated activities & dispersed resources*** | | | | | | |
| Regulated, dispersed camping | | None | | None | | None |
| Regulated, campground camping | | None | | None | | None |
| Trail use | | None | | None | | None |
| Trail ATV use | | None | | None | | None |
| ***Input nodes: regulated activities & concentrated resources*** | | | | | | |
| Regulated, dispersed camping | | None | | Uncommon | | Uncommon |
| Regulated, campground camping | | High | | None | | High |
| Trail use | | High | | None | | High |
| Regulated bear-viewing | | High | | Low | | None |
| Regulated angling – spatial/temporal | | Low | | None | | None |
| Regulated angling – fish handling | | High | | None | | High |
| ***Input nodes: unregulated activities & concentrated resources*** | | | | | | |
| Unregulated, dispersed camping | | None | | None | | Low |
| Unregulated, campground camping | | None | | None | | High |
| Unregulated bear-viewing & photography | | None | | Low | | High |
| Unregulated angling – spatial/temporal | | None | | Low | | None |
| Unregulated angling – fish handling | | None | | None | | High |
| ***Input nodes: winter recreation*** | | | | | | |
| Non-motorized winter recreation | | None | | None | | Common |
| Motorized winter recreation | | Uncommon | | None | | None |
| ***Other input nodes*** | | | | | | |
| Other hunting/non-winter trapping | | None | | None | | Low |
| Brown bear harvest | | None | | None | | Sustained yield |
| Unreported human-caused mortality | | Not_influential | | Not_influential | | Not_influential |

Table H. Sensitivity results, reported as variance reduction, for the output nodes of reproduction, cub survival, and adult reproduction given all recreational activity input nodes are in an “unknown” state (i.e. probabilities are distributed evenly among all states).

| Parent node | Reproduction | Cub survival | Adult survival |
| --- | --- | --- | --- |
| *Unregulated activities & dispersed resources* |  |  |  |
| Unregulated hiking | 0.02715 | 0.06240 | 0.02362 |
| Unregulated ATV use | 0.1908 | 0.4395 | 0.1663 |
| Unregulated, dispersed camping | 0.03188 | 0.07331 | 0.02772 |
| Unregulated, campground camping | 0.04521 | 0.1040 | 0.03934 |
| Non-motorized water recreation | 0.007406 | 0.01690 |  |
|  |  |  |  |
| *Regulated activities & dispersed resources* |  |  |  |
| Regulated, dispersed camping | 0.002978 | 0.006620 | 0.002674 |
| Regulated, campground camping | 0.002996 | 0.006679 | 0.002689 |
| Trail use | 0.01761 | 0.03842 | 0.01563 |
| Trail ATV use | 0.1175 | 0.2537 | 0.1037 |
|  |  |  |  |
| *Regulated activities & concentrated resources* | | | |
| Regulated, dispersed camping | 0.03030 | 0.05564 | 0.02067 |
| Regulated, campground camping | 0.03998 | 0.07343 | 0.02727 |
| Trail use | 0.03998 | 0.07343 | 0.02727 |
| Regulated bear-viewing | 0.02671 | 0.04907 | 0.01824 |
| Regulated angling – spatial/temporal | 0.02671 | 0.04907 | 0.01824 |
| Regulated angling – fish handling | 0.05953 | 0.1094 | 0.04063 |
|  |  |  |  |
| *Unregulated activities & concentrated resources* | | | |
| Unregulated, dispersed camping | 0.4207 | 0.5527 | 0.2462 |
| Unregulated, campground camping | 0.8084 | 1.039 | 0.4707 |
| Unregulated bear-viewing & photography | 0.4572 | 0.5965 | 0.2662 |
| Unregulated angling – spatial/temporal | 0.4572 | 0.5965 | 0.2662 |
| Unregulated angling – fish handling | 0.6881 | 0.9000 | 0.4014 |
|  |  |  |  |
| *Winter recreation* |  |  |  |
| Non-motorized winter recreation | 0.009688 | 0.07793 | 0.008104 |
| Motorized winter recreation | 0.009688 | 0.07793 | 0.008104 |
|  |  |  |  |
| *Other:* |  |  |  |
| Other hunting/non-winter trapping | 0.1551 | 0.4000 | 0.4085 |
| Brown bear harvest | - | - | 26.1 |
| Unreported human-caused mortality | - | - | 4.483 |
|  |  |  |  |
| *Intermediate nodes* |  |  |  |
| Displacement from low quality habitat | 3.94 | 9.527 | 2.657 |
| Displacement from high quality habitat | 30.75 | 56.83 | 14.83 |
| Nutritional intake | 49.18 | 92.11 | 23.72 |
| Energetic costs | 27.77 | 41.45 | 21.04 |
