# Supplementary material for: Impacts of Human Recreation on Brown Bears (Ursus arctos): A Review and New Management Tool
Source: PLoS One. 2016 Jan 5;11(1):e0141983. doi: 10.1371/journal.pone.0141983 (PMC4701408; doi:10.1371/journal.pone.0141983)

**S4 File.** User’s manual for the Bayesian network model examining the impacts of human recreational activities on Alaskan brown bears.

The Bayesian network model (BNM) was developed as a tool for managers to evaluate the potential impacts of recreational activities on Alaskan brown bears in a manager’s local jurisdiction. BNMs are influence diagrams that link variables (child and parent nodes) with conditional probabilities (Fig. 1). We developed a BNM using the modeling shell Netica (vers. 4.16, Norsys, Inc., Vancouver, British Columbia) and the software is necessary to run this model. The BNM was based on a panel that combined knowledge from five experts with specific knowledge of Alaskan brown bears, representing state and federal agencies in Alaska (S. Farley, K. Rode, G. Hilderbrand, C. Jorgensen, and J. Wilder) to establish the model structure and populate the probability tables. Due to the lack of empirical data to include directly in the model, experts used the literature review, compiled for their review, to inform their model decisions. The model was structured by the panel such that impacts on several aspects of bear biology can be gauged under all combinations of human recreational activities. The model can be run by a manager by determining the recreational activities that occur within their jurisdiction (e.g. Katmai National Park or Kenai-Russian River Area) in potential bear habitats (i.e., the recreation occurs when bears may be present in the area) during a given year. The model can also be used to vary the conditions under management control to see how those changes would influence the result.

Definitions and states for all model nodes can be found in Table S2.1. Nodes define the types of recreational activities and the conditions under which they occur. The states define the level of occurrence for that recreational activity and are measured as the number of annual user days or user nights per square mile to account for variation in size of jurisdiction (Table S2.2). Recreational activities (input nodes; turquoise nodes in Fig. 1) are combined in summary nodes (blue nodes in Fig. 1) based on if the activities are regulated or unregulated and if they occur in habitat containing concentrated (e.g., salmon or salt-marsh meadows) or dispersed (e.g., moose or cow parsnip) food resources for bears. Berries may be considered as either a dispersed or concentrated food resource by managers depending on the concentration on the landscape, type of berry, and size and extent of the berry crop. For regulated recreational activities represented in the model, we assumed a high compliance to regulations from users regardless of mechanisms for enforcement. Regulations are defined as guidelines, agency policies, and those regulations that have statutory authority relative to recreational activities that can influence bear behavior. Such regulations could include food storage, staying to a trail or defined area, camping within designated sites, bear-resistant storage of fish, or temporal or spatial use of areas important to bears (See Node descriptions, Table S2.1 below). If regulations exist but are unenforced, then the recreation should be included in the model as “unregulated.” Unregulated recreational activities include those that do not occur under regulation and those for which regulations are unenforced.

Some recreational activities were not included in the above summary nodes because of differences in the types of impact. Winter recreational activities were summarized into a separate summary node to denote their impact on bears during the denning season. Brown bear harvest and other hunting and non-winter trapping were not included in summary nodes of other recreational activities because their impacts differ from most other recreational activities. Bear hunting and other hunting input nodes include subsistence and sport hunting because they often both occur simultaneously and the biological impacts on bears cannot be differentiated.

The mechanisms of impact (intermediate nodes; tan nodes in Fig. 1) included displacement from high and low quality habitat, energetic costs, and nutritional intake. We defined high quality habitats as those that contain concentrated food resources (e.g., salmon or salt-marsh meadows) and low quality habitats as those containing dispersed food resources (e.g., moose or cow parsnip). Displacement of bears was defined as either the temporal or spatial avoidance of humans resulting in bears leaving the immediate area. Long-term displacement was defined as bears avoiding the resource for the duration that the resource is available for that year. The intermediate nodes of displacement from low and high quality habitat are discrete and measured by the probability of being displaced. Output nodes (green nodes in Fig. 1) are cub survival, reproduction, and adult survival. The intermediate nodes of nutritional intake and energetic costs and the output nodes of cub survival, reproduction, and adult survival are all continuous and are measured by percent increase or decrease over baseline (i.e. normal background levels).

When running the model, managers should consider each recreational activity in the model (input nodes) and identify the user level (how many people averaged per square mile for the active bear season) for each that occurs in their area. The modelers realize that some recreational activities (angling) or nodes (concentrated food resources) do not occur consistently across the jurisdictional area. In those cases, the manager needs to estimate the overall use of the recreational activity and portray it on an annual timeline, estimating human use on a per square mile basis. In the BNM, the manager selects the levels of recreational activities by clicking on one level in each recreational activity input node. If a manager is completely uncertain about the occurrence of a recreational activity then they should leave it in its prior (default uniform distribution) state. If they can eliminate 1 or more states, then they should set those states to zero and evenly distribute the probabilities among the remaining states (by opening the table for that input node). For example, if trail hiking is known to occur but the level is unknown then they should set “none” to 0% and distribute the probability over the remaining three states of low, medium, and high, assigning 33.3% to each (Figure S3.1).

Some recreational activity inputs may occur across management agencies and under multiple management regimes. The Kenai-Russian River Management Area (KRRMA) is a good example of this. Although a manager may be applying the model to an area such as the KRRMA, brown bear harvest is managed at the GMU level in Alaska and that is the data necessary for the model input. Both the USDA Forest Service and US Fish and Wildlife Service share management of the KRRMA and as a result, different regulations apply to different sides of the river. The entire KRRMA was considered one unit in this example and results were averaged across the active bear season on a square mile basis.

Figure A. Example of how to input probability values for recreation activities. (a) The prior state for all nodes is uniform distribution. If a manager is completely uncertain about the occurrence of a recreational activity then they should leave the node in the default state. (b) Otherwise, to specify probability values for the states different than the default uniform distribution values, right click on the title bar of the recreation activity node and select Enter Finding / Likelihood. (c) A box pops up prompting to enter the probability value for the first state, in this example for "None (0)." Note that probabilities entered this way are in the domain [0, 1.0], so if the probability for "None (0)" is 15%, enter 0.15 as shown below. Click OK. This repeats the prompt for the next state [here, for the state "Low (>0 to<10)], and so on through all states. Be sure that all probabilities entered sum to 1.0. (d) The result is to change the prior probability values of the states based on the new values entered (in this example, the priors are now [15.0, 15.0, 50.0, 20.0] for the four states in this node. Note that the expected value (± 1 SD) at the bottom of the node has been updated to reflect the changed probabilities. Next, save the model under a new file name so that these updated values can be retrieved when the model is opened again.

a.


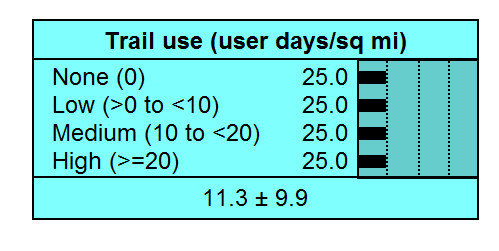


b.


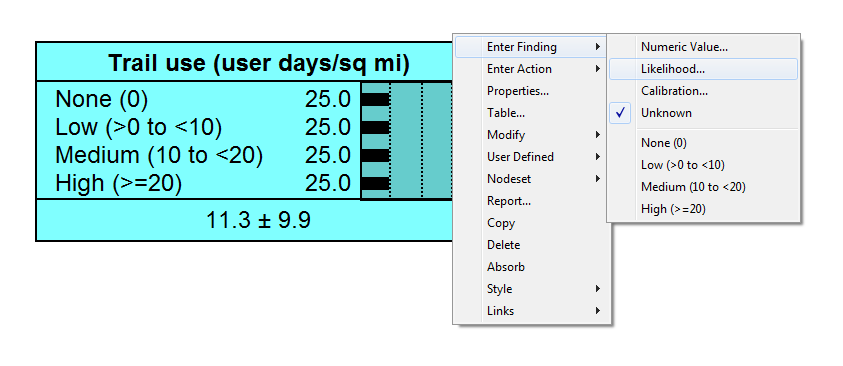


c.


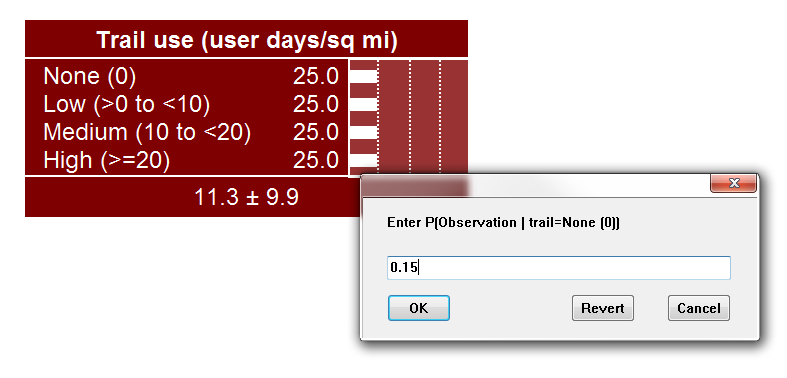


d.


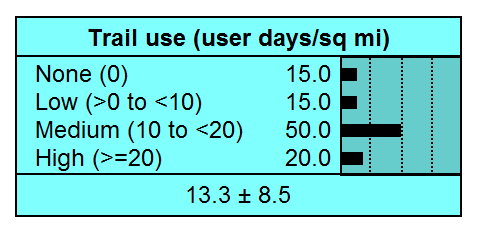

Supplement: S4 File — (DOCX) [file pone.0141983.s004.docx]
